# Luminescence and Excited-State Reactivity in a Heteroleptic Tricyanido Fe(III) Complex

**DOI:** 10.1021/jacs.3c11517

**Published:** 2023-12-29

**Authors:** Yating Ye, Pablo Garrido-Barros, Joël Wellauer, Carlos M. Cruz, Rodrigue Lescouëzec, Oliver S. Wenger, Juan Manuel Herrera, Juan-Ramón Jiménez

**Affiliations:** † Departamento de Química Inorgánica, Facultad de Ciencias, Universidad de Granada and Unidad de Excelencia en Química (UEQ), Avenida Fuente Nueva s/n, 18071, Granada, Spain; ⊥ Department of Chemistry, University of Basel, St. Johanns-Ring 19, 4056, Basel, Switzerland; § Departamento de Química Orgánica, Facultad de Ciencias, Universidad de Granada and Unidad de Excelencia en Química (UEQ), Avenida Fuente Nueva s/n, 18071, Granada, Spain; ‡ Institut Parisien de Chimie Moléculaire, CNRS, UMR 8232, Sorbonne Université, F-75252 Paris Cedex 5, France

## Abstract

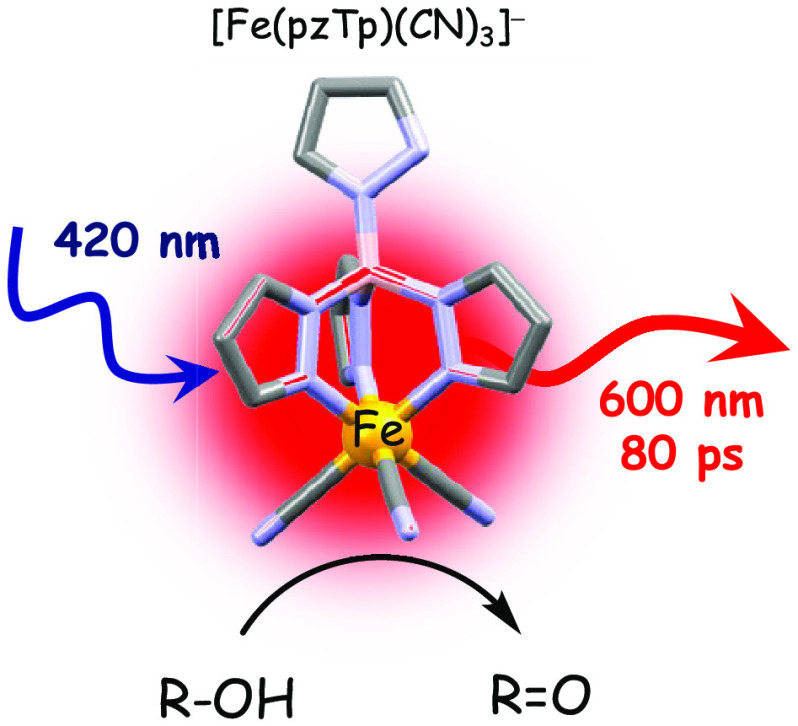

Harnessing sunlight via photosensitizing molecules is key for novel optical applications and solar-to-chemical energy conversion. Exploiting abundant metals such as iron is attractive but becomes challenging due to typically fast nonradiative relaxation processes. In this work, we report on the luminescence and excited-state reactivity of the heteroleptic [Fe^III^(pzTp)(CN)_3_]^−^ complex (pzTp = tetrakis(pyrazolyl)borate), which incorporates a σ-donating trispyrazolyl chelate ligand and three monodentate σ-donating and π-accepting cyanide ligands. Contrary to the nonemissive [Fe(CN)_6_]^3–^, a broad emission band centered at 600 nm at room temperature has been recorded for the heteroleptic analogue attributed to the radiative deactivation from a ^2^LMCT excited state with a luminescence quantum yield of 0.02% and a lifetime of 80 ps in chloroform at room temperature. Bimolecular reactivity of the ^2^LMCT excited state was successfully applied to different alcohol photo-oxidation, identifying a cyanide–H bonding as a key reaction intermediate. Finally, this research demonstrated the exciting potential of [Fe(pzTp)(CN)_3_]^−^ as a photo-oxidant, paving the way for further exploration and development of emissive Fe-based photosensitizers competent for photochemical transformations.

## Introduction

**Figure 1:**
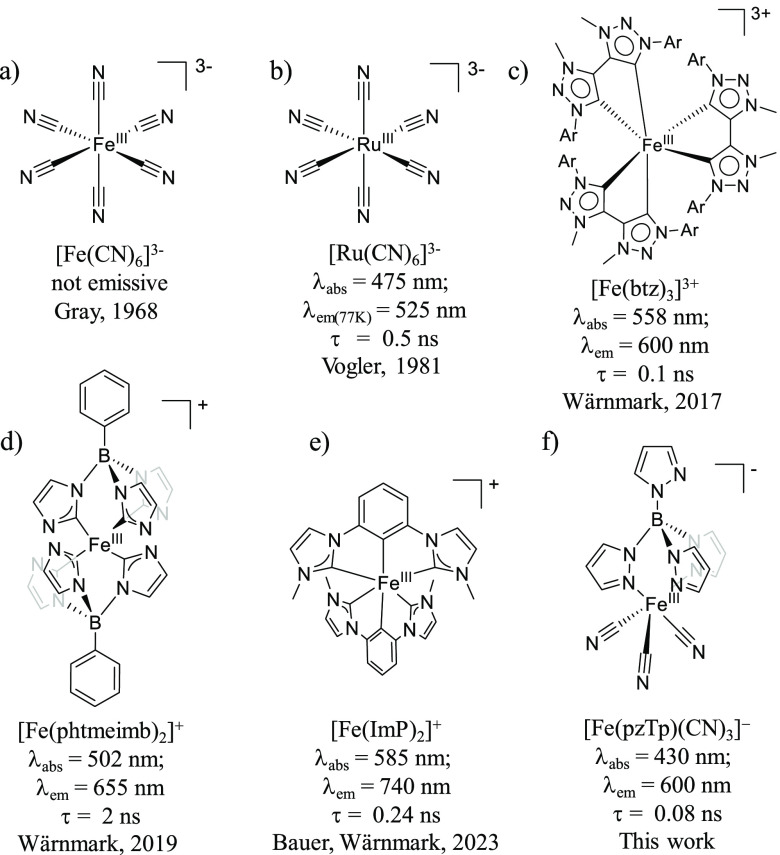
Molecular structures of the reported d^5^ emitters together with [Fe(pzTp)(CN)_3_]^−^ presented in this work.

Considering the ongoing climate and earth-resource crises, there is a growing emphasis on the development of cost-effective photoactive compounds that utilize more sustainable, earth-abundant metals for solar energy conversion and storage processes.^1−7^ Notable photosensitizers have been achieved using first-row transition metal ions, but overall progress is slow compared with the vast library of complexes based on 4d and 5d metal ions.^8,9,18,10−17^ During the last decades, special attention has been paid to Fe^II^ photosensitizers where a metal-to-ligand charge transfer (MLCT) can act as a photoactive level displaying luminescence and reactivity, similarly to precious Ru^II^ chromophores. Quite recently attention turned to Fe^III^ compounds boosting a photoactive ligand-to-metal charge transfer (LMCT).^19−27^ Early development of photofunctional d^5^ complexes was devoted to hexacyanometallates such as [M^III^(CN)_6_]^3–^ (M = Fe or Ru) where the strong ligand field splitting of the cyanide ligands generates a low-spin doublet ground state (Figure 1a,b).^28,29^ Luminescence from a ^2^LMCT excited state (λ_em_ = 525 nm; τ = 0.5 ns) for the ruthenate analogue was detected at 77 K, while no signal was found for the ferricyanide. Despite the strong ligand field splitting provided by the cyanide ligands in the iron analogue, low lying metal-centered (MC) states induce nonradiative relaxation from the potentially emissive ^2^LMCT state. This contrasts with the ruthenium analogue, which displays a low-lying ^2^LMCT state from which radiative relaxation was observed.^24^ More recently, the application of N-heterocyclic carbene (NHC) or mesoionic carbene (MIC) ligands has resulted in a conceptual breakthrough by providing a strategy to generate photoactive iron complexes. Compared to NHCs, which are strong σ-donors, the MICs possess good σ-donor and strong π-acceptor properties. Consequently, the MICs generate powerful ligand fields that destabilize dark MC states while allowing for accessible emissive charge-transfer excited states. In this context, Wärnmark and co-workers reported on homoleptic Fe^III^ complexes that harness LMCT instead of MLCT as a photoactive state (Figure 1).^24,30^ The authors showed that the [Fe(btz)_3_]^3+^ complex (btz = 3,3′-dimethyl-1,1′-bis(*p*-tolyl)-4,4′-bis(1,2,3-triazol-5-ylidene)) displays 100 ps LMCT luminescence, while the [Fe(phtmeimb)_2_]^+^ complex (phtmeimb = phenyl[tris(3-methylimidazol-1-ylidene)]borate) features rather long-lived (nanosecond range) luminescence as well as reductive and oxidative electron-transfer reactivity (Figure 1c,d).^31−34^

Subsequently, Bauer and co-workers and Wärnmark and co-workers reported on a novel example of an iron LMCT emitter (Figure 1e). The complex [Fe(ImP)_2_]^+^ (HImP = 1,1′-(1,3-phenylene)bis(3-methyl-1-imidazol-2-ylidene)) showed red emission at 740 nm with an excited-state lifetime of 240 ps.^35,36^ This exciting progress warrants further efforts to explore new design principles to generate emissive Fe-based photosensitizers that can also be competent for the photochemical transformation of more challenging substrates.

## Results and Discussion

**Figure 2:**
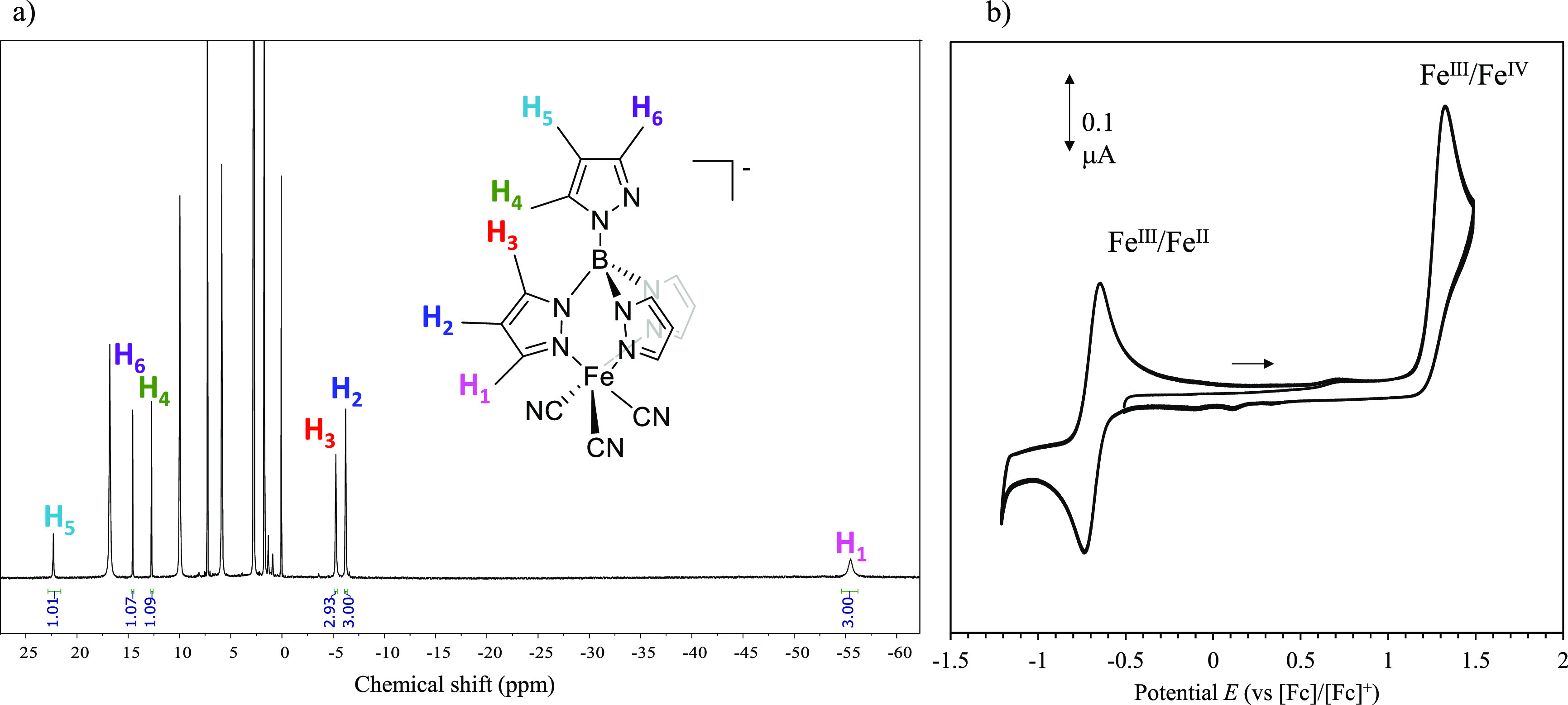
(a) ^1^H NMR of the [*n*Bu_4_N][Fe(pzTp)(CN)_3_] complex in CDCl_3_ at 300 K. (b) Cyclic voltammogram of [Fe(pzTp)(CN)_3_]^−^ (10^–3^ M) in CH_3_CN with 0.1 M [*n*Bu_4_N][PF_6_] as electrolyte at a scan rate of 100 mV/s showing formal oxidation states involved in the redox events. Glassy carbon was employed as working electrode, Pt as counter electrode, and Ag/AgOTf as reference electrode.

In this work, we disclose the photophysical and photochemical properties of the anionic heteroleptic [Fe(pzTp)(CN)_3_]^−^ complex (Figure 1f, Figure S1; see SI for synthetic details). This complex combines a rigid σ-donating trispyrazolyl borate ligand with three σ-donating and π-accepting cyanide ions, providing a strong ligand field splitting, which stabilizes low spin states (^2^T_2_ ground term).^37^ Due to the paramagnetic nature and strong magnetic anisotropy of the [Fe(pzTp)(CN)_3_]^−^ complex, it has been extensively used as metalloligand for the design and synthesis of single molecule magnets (SMMs), single chain magnets (SCMs), and photomagnetic coordination clusters, but the photophysical and photochemical properties remain unexplored.^38,39^ The negatively charged pzTp^–^ ligand coordinates in a facial (*fac*) mode to Fe^III^ together with three cyanide ligands that complete the coordination sphere (Figure 2a). The Fe–C and Fe–N bond lengths amount to 1.919(5) and 1.976(9) Å, which are typical for these kinds of heteroleptic complexes (Table S1 first column).^37^ Deviation from an ideal octahedral geometry has been calculated using the expression ∑ = ∑_*i*=1_^12^ = |90 – ϕ*i*|, where ϕ*i* are the cisoid bond angles (Table S1 first column) and amount to 23°, which demonstrates the small distortion compared to a perfect octahedron geometry. This will favor the overlapping of metal and ligand orbitals, increasing the ligand field splitting. The paramagnetic nature of [Fe(pzTp)(CN)_3_]^−^ was evidenced by ^1^H NMR. As expected, the signals are strongly shifted outside the “regular” diamagnetic range due to the unpaired electron (Figure 2a). The ^1^H NMR spectrum was recorded at 300 K in CDCl_3_. The trigonal symmetry of the pzTp^–^ ligand binding the Fe^III^ generates one pyrazolyl set of signals (three signals: H1–H3), integrating for three protons each. The fourth pyrazolyl ring is expected to freely rotate, giving three additional signals integrated for one proton each (H4–H6, Figure 2a). This assignment confirms the *C*_3*v*_ symmetry of the [Fe(pzTp)(CN)_3_]^−^ complex in solution.

The cyclic voltammogram (CV) of freshly dissolved [Fe(pzTp)(CN)_3_]^−^ exhibits a single quasi reversible Fe^III^/Fe^II^ reduction wave at *E*°_1/2_ = −0.70 V vs [Fc]/[Fc]^+^ (Figure 2b). This complex is reduced at a significantly higher potential than the homoleptic homologues [Fe(phtmeimb)_2_]^+^ (*E*°_1/2_ = −1.16 V vs [Fc]/[Fc]^+^) and [Fe(CN)_6_]^3–^ (*E*°_1/2_ = −1.397 V vs [Fc]/[Fc]^+^) and also than [Fe(ImP)_2_]^2+^ (*E*°_1/2_ = −1.16 V vs [Fc]/[Fc]^+^), which indicates the stronger donor properties of mesoionic ligands and six-coordinated cyanide ligands and promises a more oxidizing excited state. On the other hand this redox potential is comparable with [Fe(btz)_3_]^3+^ (*E*°_1/2_ = −0.58 V vs [Fc]/[Fc]^+^) and [Fe(bipy)(CN)_4_]^−^ (*E*°_1/2_ = −0.63 V vs [Fc]/[Fc]^+^).^40^ On the anodic scan, an irreversible wave at *E*°_1/2_ = 1.37 V vs [Fc]/[Fc]^+^ has been detected and associated with the Fe^III^/Fe^VI^ process. Upon reverse cycling, some reduction peaks appear between 0 and 0.5 V, which are attributed to the reduction of side products generated at 1.37 V.

**Figure 3:**
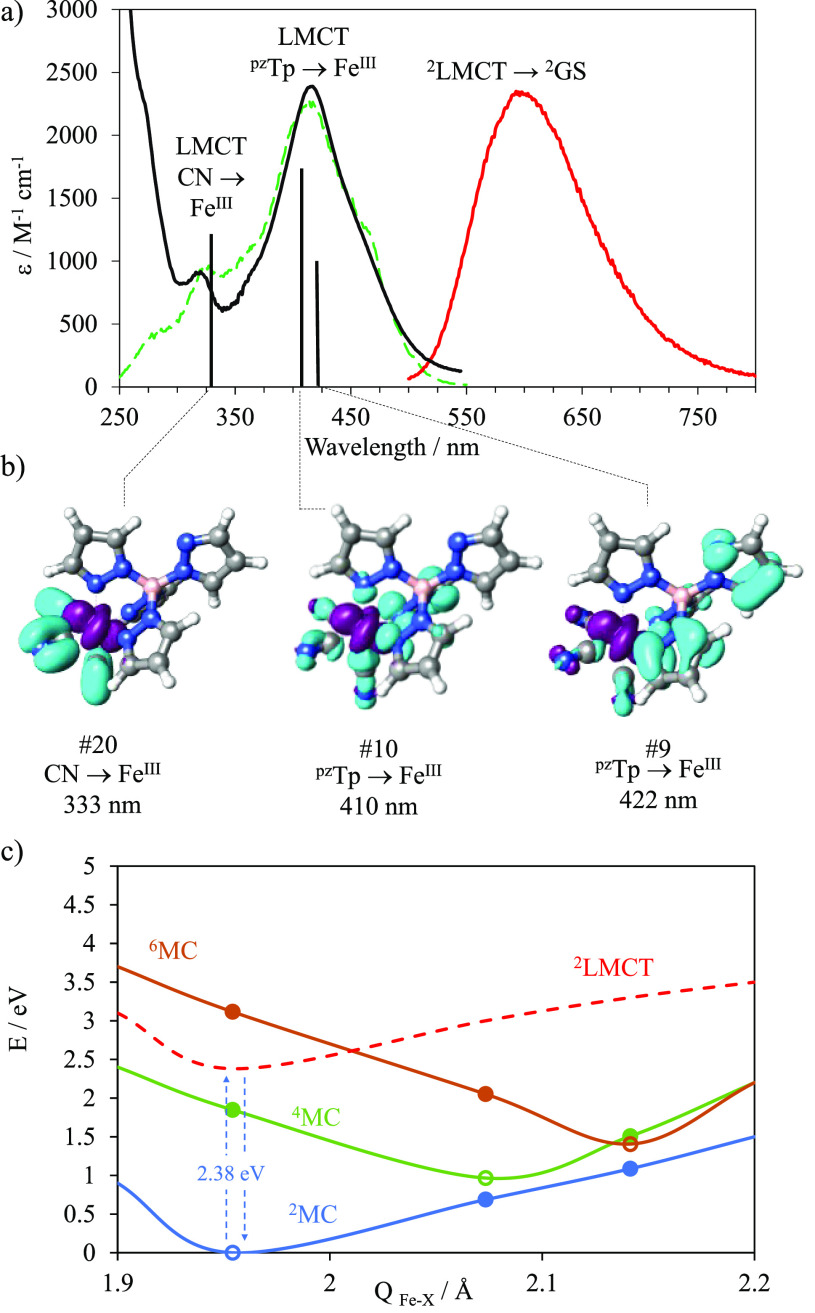
(a) Absorption spectrum (black line), emission spectrum upon excitation at 420 nm (red line), and excitation spectrum (dashed green line). Vertical lines correspond to calculated transitions by TDDFT. (b) Electron density difference maps (EDDM) of the most intense calculated electronic transitions for [Fe(pzTp)(CN)_3_]^−^. Blue: density loss; purple: density gain. (c) Potential energies and Fe–X equilibrium bond lengths (*Q*) for relevant electronic states of [Fe(pzTp)(CN)_3_]^−^ and potential surfaces (lines) drawn through the energy of each state at the geometry of the two other states (circles). The ^2^LMCT surface was extrapolated from the ground-state (GS) shape and experimental energy.

The visible absorption spectrum of [Fe(pzTp)(CN)_3_]^−^ in chloroform displayed a broad band with a maximum at 420 nm (ε = 2352 M^–1^ cm^–1^) and a smaller band located at 318 nm (ε = 902 M^–1^ cm^–1^) (Figure 3a). According to time-dependent density functional theory (TDDFT), both bands are mainly associated with LMCT transitions (Figure S5). The higher energy band corresponds to a charge transfer from the π orbitals of the cyanides to the d orbitals of the iron center (CN → Fe^III^), calculated at 333 nm (Figure 3b). The broad band with a maximum at 420 nm is an admixture of two main charge-transfer transitions from the pzTp^–^ π orbitals to the iron center (pzTp → Fe^III^), calculated at 422 and 411 nm (Figure 3b). Noteworthy, the absorptivity molar coefficient of the heteroleptic compound has a larger value compared to the [Fe(CN)_6_]^3–^ (ε_420 nm_ = 1200 M^–1^ cm^–1^),^29,41^ which indicates a more allowed character of the LMCT in the [Fe(pzTp)(CN)_3_]^−^ complex. This is likely due to a lowering in symmetry on going from *O*_*h*_ → *C*_3*v*_ together with additional vibronic perturbations introduced by the tetrakis(1-pyrazolyl)borate ligand.

Upon excitation at 420 nm, within the highest LMCT band, a broad emission band with a maximum at 600 nm and full width at half-maximum (fwhm) of 3000 cm^–1^ was detected in chloroform at ambient temperature (Figure 3a; solid red line). The excitation spectrum perfectly matches the absorption spectrum, which provides unambiguous evidence that the luminescence originates from the [Fe(pzTp)(CN)_3_]^−^ complex (Figure 3a; dashed green line). To the best of our knowledge, this is the first reported example of luminescence arising from an iron-cyanido molecular complex. The quantum yield was measured using a comparative method with [Ru(bipy)_3_]^2+^ as a reference and amounts to 0.02%. This value is significantly lower than the 2.1% reported for [Fe(phtmeimb)_2_]^+^ but comparable to the 0.03% reported for the [Fe(btz)_3_]^3+^ complex. DFT calculations (see SI for details) reproduced accurately the geometry obtained by XRD for the ^2^MC state (Figures S2, S3 and Table S1) and showed that the ^4^MC state is stabilized (1.85 eV) with respect to the ^2^LMCT state (*E*_0–0_ = 2.38 eV = 19 230 cm^–1^) (Figure 3c); similar results were obtained after CASSCF(5,5)/FIC-NEVPT2 calculations (Table S2 and Figure S4). This situation contrasts with that observed in the above-mentioned related complexes,^31,32,35^ where the ^4^MC state is slightly higher in energy than the ^2^LMCT state (calculated from the intersection of the normalized emission and absorption spectra, *E*_00_). Despite this unfavorable situation, luminescence from the ^2^LMCT state in [Fe(pzTp)(CN)_3_]^−^ could be detected, which likely competes with the nonradiative relaxation through dark MC states and should be responsible for the low value of measured quantum yield.

The excited-state evolution dynamics was characterized by the simultaneous decay of transient UV–vis absorption and time-resolved photoluminescence in CHCl_3_ at room temperature. Similar excited-state lifetimes of 80 ± 2 ps were obtained from both types of measurements (Figures S7, S8). The observable transient absorption difference spectra support the charge-transfer nature of the photoactive excited state. As stated above, the facile conversion to a lower energy MC state is likely responsible for the short ^2^LMCT excited-state lifetime. From the excited-state lifetime and luminescence quantum yield the radiative ^2^LMCT → ^2^GS decay rate (*k*_rad_) was estimated to be 2.63 × 10^6^ s^–1^, which is in line with the allowed character of the radiative transition. These figures of merit are in excellent agreement with those obtained previously for the [Fe(btz)_3_]^3+^ complex.^32^

Despite the short excited-state lifetime, [Fe(pzTp)(CN)_3_]^−^ displays a very appealing excited redox potential of *E°*(Fe^*III/II^) = 1.68 V vs [Fc]/[Fc]^+^ (eq 1; *E*_00_ is the crossing point between the absorption and emission spectra), which points to its competence as a photo-oxidant.
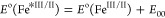
1

**Figure 4:**
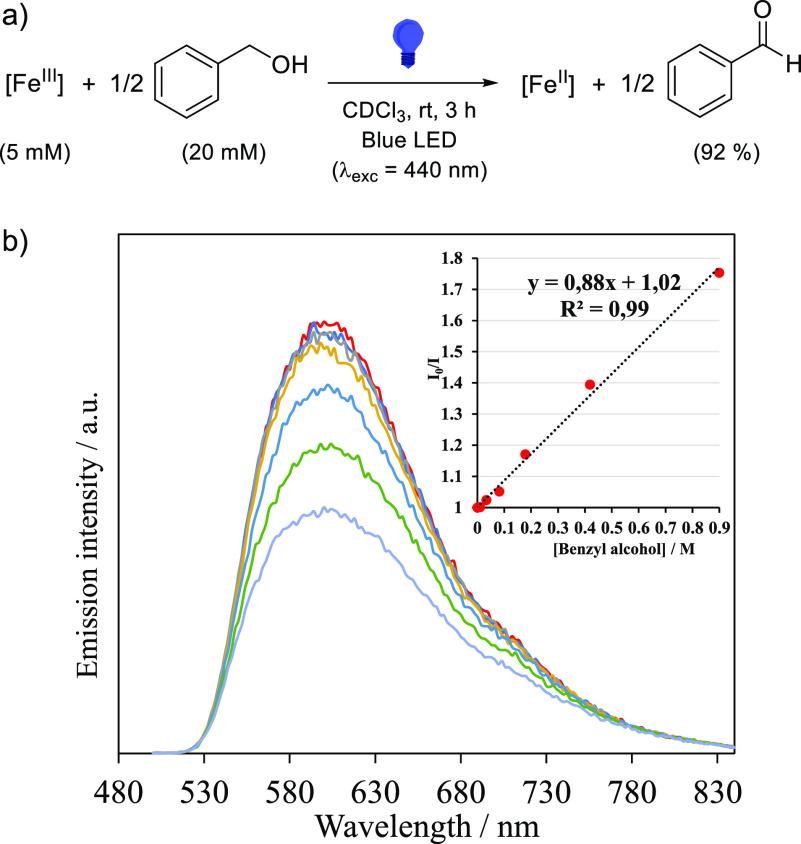
(a) Reactivity of the ^2^LMCT excited state of [Fe(pzTp)(CN)_3_]^−^ ([Fe^III^]) toward oxidation of benzyl alcohol with the crude NMR yield. (b) Emission quenching monitored by steady-state emission spectra for increasing concentrations of benzyl alcohol. Stern–Volmer plot for steady-state intensity from quenching experiments (inset). A quenching rate constant *k*_ET_ of 1.1 × 10^10^ M^–1^·s^–1^ is extracted from this data set.

This value is similar to the redox potential associated with the ligand oxidation obtained from cyclic voltammetry, consistent with LMCT excitation that generates an oxidized ligand (Figure S26). Thus, the excited-state reactivity of this complex was studied toward the photo-oxidation of alcohols as challenging model substrates with relevance in synthetic methodologies and energy applications.^42−45^ Benzyl alcohol (BnOH) provides a suitable platform to initially test this reactivity due to its accessible oxidation (*E°* = 1.6 V vs [Fc]/[Fc]^+^, Figure S23). Irradiating a solution of [Fe(pzTp)(CN)_3_]^−^ (0.1 mM) and excess BnOH (15 mM) in chloroform with a blue LED (λ_exc_ = 440 nm) leads to the decrease of the LMCT band at 420 nm (Figure S9) of the Fe^III^ species and the concomitant increase of a band at 350 nm. The latter is attributed to an MLCT process from the Fe^II^ homologue, as supported by TD-DFT (Figure S6) and previous work.^46^ The product of this reaction has been characterized via ^1^H NMR after 3 h of irradiation of a solution containing 5 mM [Fe(pzTp)(CN)_3_]^−^ and 20 mM BnOH. The spectrum reveals the formation of benzaldehyde from a net 2 e^–^ oxidation in 92% yield relative to the maximum conversion of 2.5 mM considering the concentration of the Fe complex and the stoichiometry of the reaction (yields reported here are NMR yields; Figure 4a and Figure S13). The NMR spectrum also shows the appearance of new diamagnetic peaks that are associated with the aromatic protons of the Fe^II^ species. The control reaction in the absence of BnOH failed to produce any changes in the UV–vis spectrum, and irradiation of BnOH in the absence of [Fe(pzTp)(CN)_3_]^−^ yields no oxidation product (Figure S10).

Using 1-phenylethanol, with a higher oxidation potential of 1.7 V vs [Fc]/[Fc]^+^ (Figure S24), results in similar experimental observations with the reduction of [Fe(pzTp)(CN)_3_]^−^ to the corresponding Fe^II^ species (Figure S9). Acetophenone was formed in 46% yield as the oxidation product under conditions similar to those previously observed; the decreased yield might reflect the lower driving force for the oxidation of a phenyl versus benzylic alcohol (Figure S14). Running the photo-oxidation for 6 h instead affords 84% of acetophenone (Figure S15). In addition, analogous spectroscopic characterization supports the oxidation of isopropyl alcohol, iPrOH, as an example of an aliphatic alcohol (Figure S9). Irradiation of the reaction mixture during 6 h using a higher excess of 2-propanol (50 mM) followed by NMR analysis revealed the formation of acetone in 38% yield (Figure S16). This oxidation is moderately uphill (Δ*G*^o^ = 4.6 kcal·mol^–1^), presumably enabled by stabilizing interactions (*vide infra*) and the forward steps to generate acetone. Control reactions under dark conditions failed to produce any product (Figures S17–S19).

While previous results confirm competent photo-oxidation reactivity, the low value for the excited-state lifetime suggests the interplay of preassociation interactions with the substrate. This issue was probed spectroscopically. Addition of the alcohol substrates to a solution containing [Fe(pzTp)(CN)_3_]^−^ produced a slight redshift in the LMCT band observed at 333 nm, indicative of a weak cyanide–alcohol interaction affecting the coordination sphere of the iron center (Figure S11). The corresponding IR spectrum further corroborated this observation by revealing a significant shift in the CN stretching peak toward higher energy (Figure S12). This interaction is consistent with hydrogen bonding between the alcohol group and the CN ligand. Therefore, the role of the latter goes beyond defining the electronic structure and absorption properties of [Fe(pzTp)(CN)_3_]^−^ by prearranging the reactive adduct and providing a means to exploit the strong oxidant character of the excited state.

The kinetics of these photooxidation processes were interrogated by Stern–Volmer studies (Figures 4, S20, and S21). The three substrates led to linear fits with different slopes (*K*_SV_), reflective of the different oxidation kinetics. Using the measured lifetime of the [Fe(pzTp)(CN)_3_]^−^ excited state (τ_0_) to calculate the rate of the electron transfer (*k*_ET_) assuming a dynamic quenching provides values on the order of 1.7 × 10^9^ to 1.1 × 10^10^ M^–1^·s^–1^, slightly above the diffusion-limited rate constant. In addition, plotting ln(*k*_ET_) versus the driving force for the oxidation reaction (−Δ*G*_ET_) leads to a linear correlation, in agreement with the low driving force regime of the Marcus theory (Figure S22),^47^ but with a Brønsted coefficient of 0.136 ± 0.004 estimated from the slope, significantly lower than the theoretical 0.5.^48−50^ These observations are instead consistent with a predominant static quenching involving the evidenced hydrogen-bonded adducts, which affords a pathway for excited-state electron transfer with short lifetimes of 80 ps. For such a static quenching event, *K*_SV_ becomes the equilibrium constant of the hydrogen-bonded (*K*_HB_) species,^51^ which is calculated to be 0.88, 0.42, and 0.13 M^–1^ for benzyl alcohol, 1-phenylethanol, and isopropanol, respectively. Time-resolved luminescence quenching experiments were hampered by slow reoxidation of Fe^II^ to Fe^III^, and therefore could not be conducted with a high repetition rate laser in time-correlated single photon counting experiments.

In conclusion, this work focused on the study of the optical and photochemical properties of the anionic heteroleptic [Fe(pzTp)(CN)_3_]^−^ complex. The heteroleptic design in this complex provides a good balance between relatively milder Fe^III^/Fe^II^ redox potentials and sufficiently destabilized MC states to allow for emission and photoreactivity as compared to the homoleptic analogues. The complex displayed absorption bands mainly associated with LMCT transitions and a broad emission band originating from the lowest ^2^LMCT excited state. The quantum yield of the luminescence was found to be relatively low, likely due to nonradiative relaxation through metal-centered states, but the complex turned out to be a strong photo-oxidant. The excited-state reactivity was tested with alcohols as challenging model substrates, and successful stoichiometric photo-oxidation reactions were observed. We have found a key role of the CN ligand to enable photo-oxidation of alcohol substrates via H-bonding preassociation, illustrating a potential tool to leverage the unique redox properties of photosensitizers based on earth-abundant metals that typically feature short lifetimes. This research paves the way for further exploration and development of heteroleptic emissive Fe-based photosensitizers competent for catalytic photochemical transformations.

## Supporting Information Available

Experimental details; theoretical calculations; Stern–Volmer experiments; CV of substrates; absorption spectra and Marcus analysis; transient UV–vis absorption data and time-resolved experiments (PDF)
