# Supplementary material for: Luminescence and Excited-State Reactivity in a Heteroleptic Tricyanido Fe(III) Complex
Source: J Am Chem Soc. 2023 Dec 29;146(1):954–60. doi: 10.1021/jacs.3c11517 (PMC10786067; doi:10.1021/jacs.3c11517)
Supplement: Supplementary file 1 — ja3c11517_si_001.pdf [file ja3c11517_si_001.pdf]

Luminescence and Excited State Reactivity in a Heteroleptic Tricyanido Fe(III) Complex

Yating Ye,[†] Pablo Garrido-Barros,[*,†] Joël Wellauer,[⊥] Carlos M. Cruz,[*,§] Rodrigue Lescouëzec,[‡] Oliver S. Wenger,[⊥] Juan Manuel Herrera,[†] Juan-Ramón Jiménez[*,†]

† Departamento de Química Inorgánica, Facultad de Ciencias, Universidad de Granada and Unidad de Excelencia en Química (UEQ), Avda. Fuente Nueva s/n, 18071, Granada, Spain. E-mail: jrjimenez@ugr.es; pgarridobarros@ugr.es

⊥ Department of Chemistry, University of Basel, St. Johanns-Ring 19, 4056, Basel, Switzerland

§ Departamento de Química Orgánica, Facultad de Ciencias, Universidad de Granada and Unidad de Excelencia en Química (UEQ), Avda. Fuente Nueva s/n, 18071, Granada, Spain. Email : cmorenoc@ugr.es

‡ Institut Parisien de Chimie Moléculaire, CNRS, UMR 8232, Sorbonne Université, F-75252 Paris cedex 5, France

Supporting Information

(44 pages)

Table of contents

1. **Experimental part…………………………………………………………………... S2**
2. **Synthetic details……………………………………………………………………... S3**
3. **Theoretical calculations: structural and electronic analysis ………………………...S6**
4. **Transient absorption and time-resolved measurements ……………………………S21**
5. **Absorption spectra……………………………………………………………………S23**
6. **FTIR spectra…………………………………………………………………...……….S26**
7. **Photochemical studies………………………………………………………………S27**
8. **Geometries……………………………………………………………………………..S37**
9. **References……………………………………………………………………………...S43**

1. Experimental part

Elemental analyses were carried out on a Fisons-Carlo Erba analyser model EA 1108. Absorption spectra in chloroform solution were recorded using a Jasco Cary (Agilent Technologies) spectrometer (quartz cell path length 1 cm). IR spectra were recorded on a Bruker Tensor 27 spectrophotometer by using ATR detection. NMR spectra were recorded on a Bruker Ascend 400 MHz spectrometer, with chemical shifts being reported relative to the residual solvent signal. Emission and excitation spectra were measured on a UV-VIS-PTI QuantaMaster™ 8000 spectrofluorometer equipped with a Picosecond Photon Detector (230-850nm, PPD-850, HORIBA Scientific) and a continuous Xenon Short Arc Lamp (190-2000nm, USHIO). All the spectra (emission and excitation) were corrected with real-time correction functions. Luminescence quantum yield is reported relative to $[Ru(bpy)_3]^{2+}$ in aerated H_2O (ϕ = 4.0%; λ_{exc} = 440 nm).[1] Quantum yields are thus determined from the ratio of integrated peak areas, with an assumed experimental uncertainty of ±10%. Cyclic Voltammetry measurements were conducted in a one-compartment three-electrode cell for 0.15 mM analyte solutions in dry, deaerated MeCN. nBuN$_4$PF$_6$ was utilized as the supporting electrolyte with a solution concentration of 0.1 M. Glassy carbon (3 mm diameter) was employed as the working electrode while Pt wire was utilized as the counter electrode and a Ag/AgOTf (5mM) reference electrode. All potentials are measured against the Fc^+/Fc couple. The GC working electrode was polished using alumina 0.05 µm powder. Transient UV-Vis absorption spectroscopies with sub-picosecond time resolution were measured using a HARPIA-TA instrument (Light Conversion). In this experimental setup, the excitation light is generated by a PHAROS laser (Light Conversion, Yb:KGW laser, source wavelength = 1030 nm, pulse duration = ~190 fs, repetition rate = 5.0 kHz, output power = 1.0 W, pulse energy = 0.2 mJ), and the pump light wavelength (420 nm) was generated by the optical parametric amplifier ORPHEUS (Light Conversion, used ~90% of fundamental pulse). The probe light was generated by a sapphire (5 mm thickness; ~10% of the fundamental pulse was used to generate a white light super-continuum). Sample solutions (ca. 0.2 mM, absorbance = ~0.5 at the excitation wavelength) were measured in a 1 mm quartz cuvette at room temperature. Obtained transient absorption spectra were analyzed using the CarpetView software (Light Conversion). Photoluminescence lifetime studies were performed on an FLS1000 spectrometer (time-correlated single photon counting (TCSPC) technique) from Edinburgh Instruments using a

pulsed LED for excitation at 405.0 nm (Edinburgh Instruments, pulse width: 59.7 ps, linewidth <4.5 nm), where the cuvette holder was equipped with a temperature controller (TC 1, Quantum Northwest).

2. Synthetic details

All the chemicals were purchased from commercial suppliers and used without further purification.

Potassium tetrakis (1-pyrazoly) borate (KpzTp):

Potassium borohydride (3.78 g, 70 mmol) was mixed to an excess of pyrazole (23.82 g, 350 mmol) in a 50 mL round bottom flask. The solid mass was heated first at 90 °C in an oil bath about an hour. The flask was covered with an aluminum foil. Then the reaction mixture was heated to 120 °C and kept at that temperature until hydrogen evolution was ceased. The temperature of the melt was gradually raised to 185 °C and kept at that temperature until hydrogen evolution was ceased. Finally, the reaction mixture was heated to 230 °C and left overnight. The melt was cooled to 120 °C and was poured in hot toluene (100 mL) giving a white precipitate. The white solid was filtered then washed successively with hot toluene (20 mL) and hexane (15 mL), air dried; yield = 17.8 g (80 %). ATR-IR : ν (cm-1) = 1502, 1387, 1258, 1210, 1097, 1046, 819. ^{1}H-NMR (400 MHz, D_2O) : δ (ppm) = 7.60 (d, J = 1.7 Hz, 4H), 7.30 (d, J = 2.3 Hz, 4H), 6.25 (t, J = 2.1 Hz, 4H). ^{13}C-NMR (101 MHz, D_2O): δ (ppm) = 141.61 (s, 4C), 135.06 (s, 4C), 104.61 (s, 4C),

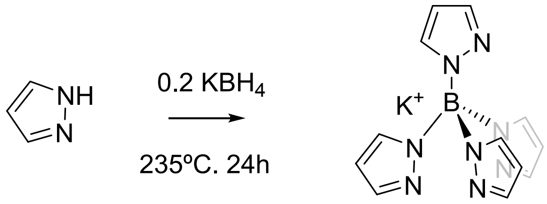

[Fe^{II}(pzTp)$_2$]:

$FeSO_4 \cdot 7H2O$ (4.17 g, 15 mmol) and KTp (7.56 g, 30 mmol) were separately dissolved in water (100 mL and 20 mL, respectively). The iron sulfate solution was added to the KTp solution, and a pinch of ascorbic acid was added to it. A purple solid was precipitate, and the reaction mixture was stirred for 15 min. After filtration, the purple solid was washed with absolute ethanol and air-

dried. Recrystallization in $CHCl_3$ gave purple crystals. Yield = 6.12 g (85%). ^{1}H-NMR (300 MHz, $CDCl_3$) : δ (ppm) = 7.64 (d, 1H, 3JH-H = 1.0 Hz NCH), 7.34 (d, 1H, 3JH-H = 1.0 Hz NCH), 6.30 (s, 1H, CCH). ^{1}H-NMR (400 MHz, $CDCl_3$) : δ (ppm) = 8.22 (s, 1H), 8.05 (d, J = 7.8 Hz, 3H), 7.10 (s, 1H), 6.69 (s, 1H), 6.50 (s, 3H). ^{13}C-NMR (101 MHz, $CDCl_3$): δ (ppm) = 152.91 (s, 1C), 141.76 (s, 3C), 135.67 (s, 4C), 109.02 (s, 1C), 106.70 (s, 3C).

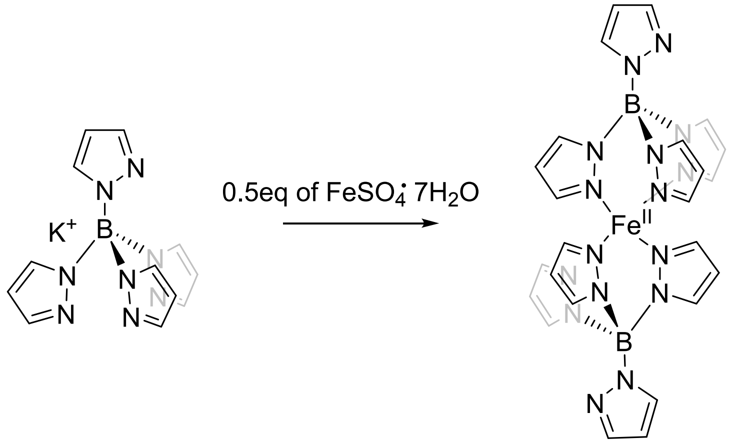

***n*NBu₄[Fe^III^(pzTp)(CN)₃]:**

$[Fe^{II}(^{pz}Tp)_2]$ (6.03 g, 12.56 mmol) and KCN (2.70 g, 41.48 mmol) were mixed in methanol (50 mL) and the resulting suspension was heated to reflux at 80 °C for 18 hours under continuous stirring. The colour of the reaction mixture changed from purple to yellowish brown. After cooling the reaction mixture down to room temperature, a yellowish-brown precipitate was obtained, filtered and air-dried. The brownish yellow solid was then washed with CH_2Cl_2 (2*25 mL) to remove the unreacted $Fe^{II}(tp)_2$. This solid (4.52 g, 10.2 mmol) in warm H_2O (600 mL; T = 40 °C) was placed into a large erlenmeyer flask (3L) covered with aluminum foil under continuous stirring. Tetrabutylammonium bromide dissolved in H_2O (50 mL) was added. Hydrogen peroxide (30 % in water) was added stepwise in 1 mL amounts until reaching a total amount of 20 mL. The solid was recrystallized from acetonitrile (200 mL) and 20 mL of water were added after filtration of the acetonitrile solution. The pure product was obtained as orange crystals. Yield: 5.62 g (80%). ATR-IR: ν = 2124 cm^{-1} (Fe^{III}-CN). Elemental analyses for: $C_{31}H_{48}BFeN_{12}\cdot 0.35H_2O$ %, found: C: 56.30, N: 7.45, H: 25.3; calculated: C: 56.26, N: 7.42, H:

25.4. ^{1}H-NMR (400.1 MHz, $CDCl_3$) δ (ppm) = 22.30 (1H), 16.80 (9H), 14.56 (1H), 12.72 (1H), 9.96 (9H), 5.87 (9H), 2.76 (9H), -5.27 (3H), -6.22 (3H), -55.49 (3H).

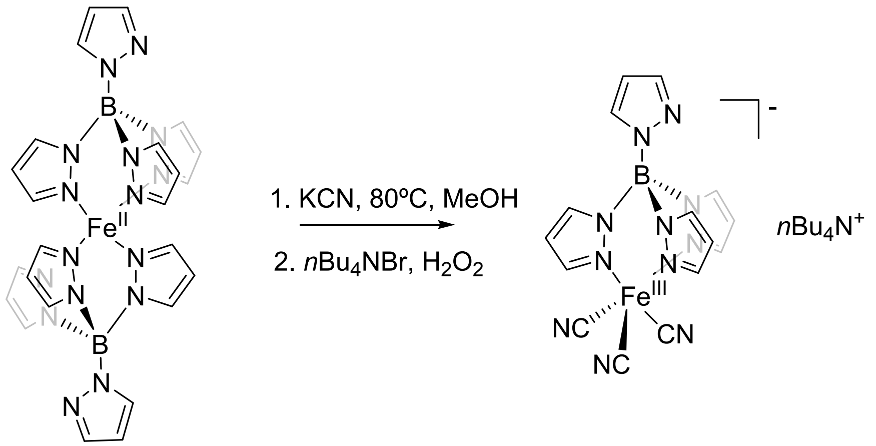

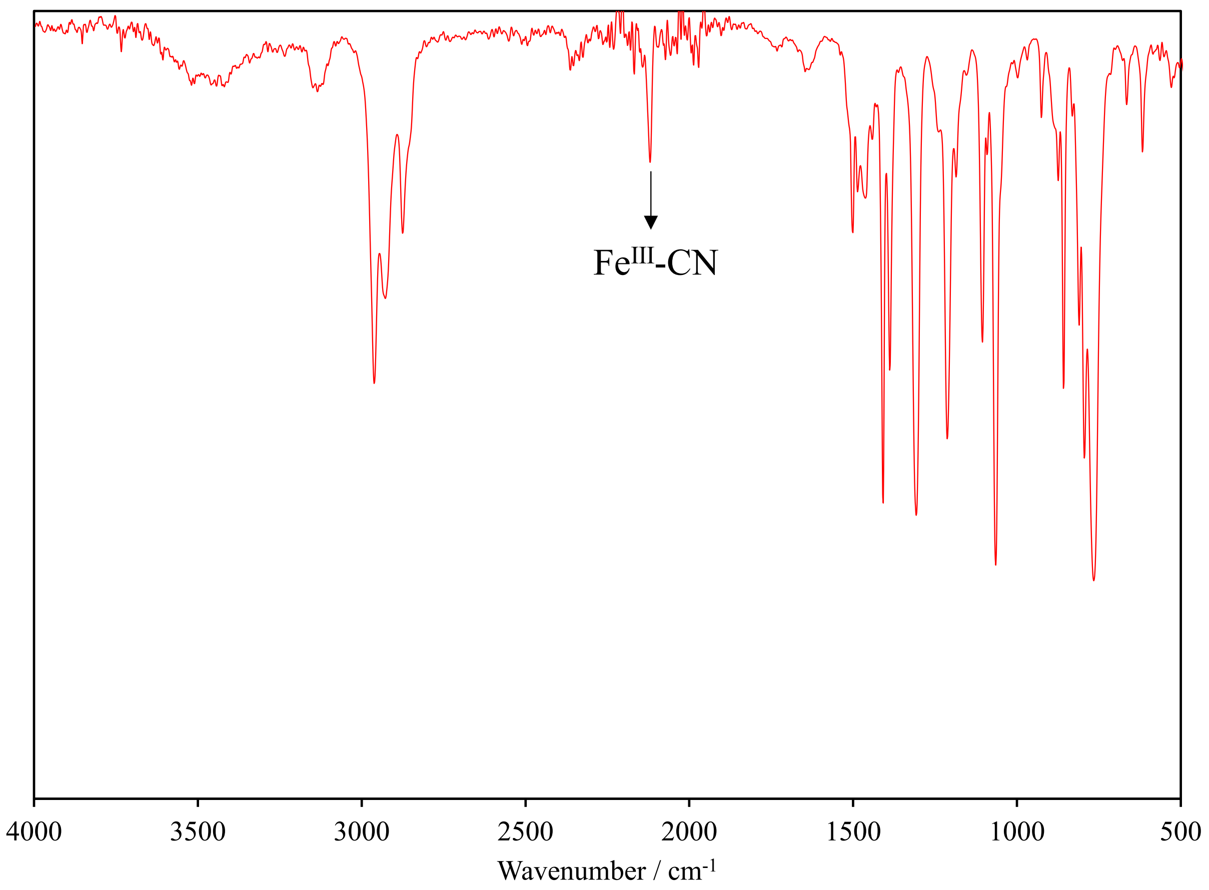

Figure S1. FTIR spectrum of the $[nNBu_4][Fe^{III}(pzTp)(CN)_3]$ at room temperature.

3. Theoretical calculations: structural and electronic analysis

The Orca[2] (version 5.0.2) software package was used to investigate the structural and electronic properties of $[Fe(pzTp)(CN)_3]^-$. Starting from the X-ray diffraction structure, the ground, $^2T_2$ state, state geometry was obtained from DFT optimizations using the unrestricted version of the Becke three-parameters exchange function in combination with the Lee-Yang-Parr correlation functional with a 15% of Hartree-Fock exchange (UB3LYP*). The Ahlrichs' polarized valence triple-ζ basis set def2-TZVPP was used for the optimization. The D3 version of Grimme's dispersion with Becke-Johnson damping (GD3BJ) was applied. Solvent effects were included via the Conductor-like Polarizable Continuum Model (CPCM) as implemented in Orca 5.0.2 with the dielectric constant of chloroform. Optimized geometries were confirmed to be stationary points by analysis of their vibrational frequencies. Tight convergence criteria were selected for the optimization step. The resolution of identity approach for the Coulomb term in combination with the chain-of-spheres approximation for the exchange term (RIJCOSX) was applied. The zero-order relativistic approximation (ZORA) was used to describe relativistic effects in all calculations. Spin density information was extracted from the optimized geometries.

Ab initio ligand field (AILF) analysis was performed over the optimized geometries using Orca version 5.0.2. The complete-active-space self-consistent field method (CASSCF) together with the fully internally contracted N-electron valence perturbation theory to second order (FIC-NEVPT2) was used, selecting only the 3d orbitals as active space (CASSCF(5,5)/FIC-NEVPT2) and using the def2-TZVPP basis set in combination with the RI-JK approximation (def2/JK as auxiliary base). 51 doublet, 21 quartet and 1 sextet roots were computed for the AILF analysis.

The 50 lowest energetic transitions were calculated by TD-DFT using the Tamm-Dancoff approximation (TDA) as implemented in Orca version 5.0.2, using the unrestricted version of the B3LYP* functional, selecting the def2-TZVPP basis set and considering solvent effects. Electronic transitions were corrected by –0.1 eV to better fit experimental results.

Optimized Geometries and Spin Densities

$[Fe(pzTp)(CN)_3]^-$ - $^2T_2$ state

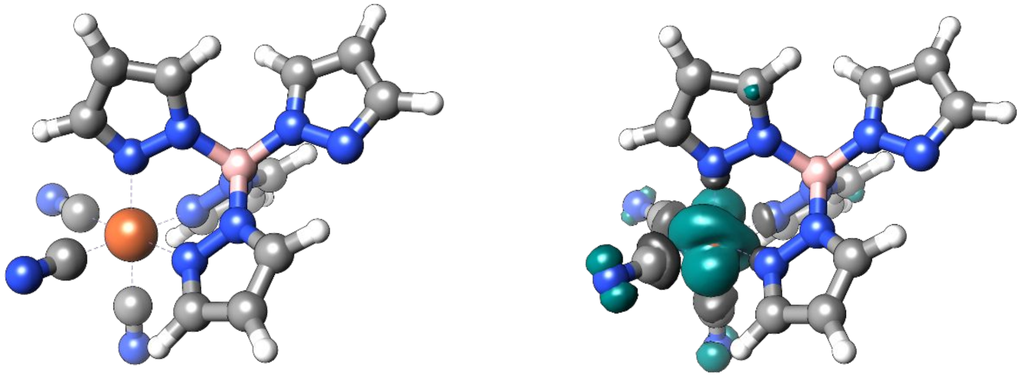

$[Fe(pzTp)(CN)_3]^-$ - $^4T_1$ state

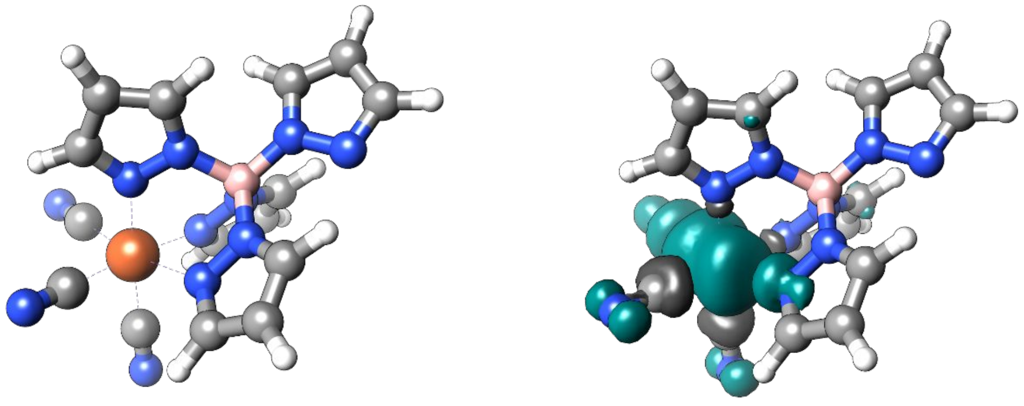

$[Fe(pzTp)(CN)_3]^-$ - $^6A_1$ state

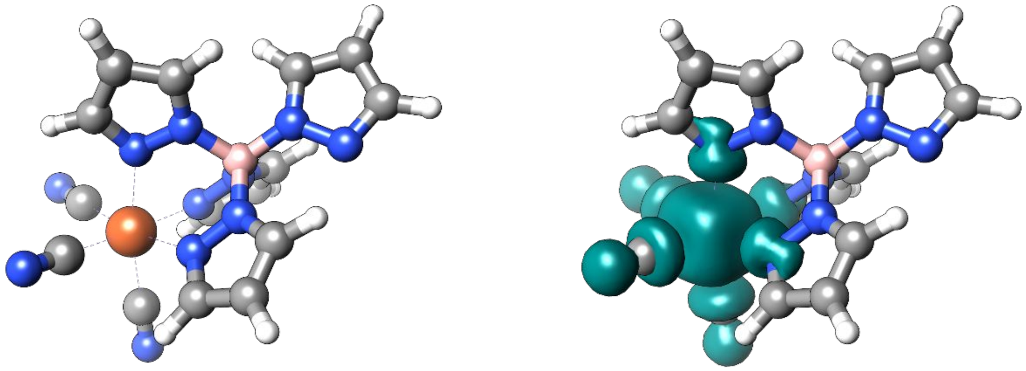

Figure S2. DFT optimized geometries and spin density maps (isoval = 0.002) of the $^2T_2$ (top), $^4T_1$ (middle) and $^6A_1$ (bottom) states of $[Fe(pzTp)(CN)_3]^{-}$. Spin density at the Fe center: 1.039476 ($^2T_2$), 2.941957 ($^4T_1$) and 4.435274 ($^6A_1$).

$[Fe(pzTp)(CN)_3]^{2-}$ - $^1A_1$ state

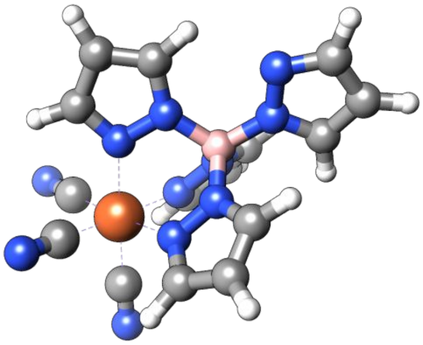

Figure S3. DFT optimized geometry of $[Fe(pzTp)(CN)_3]^{2-}$.

Table S1. Bond lengths (Å) and angles (°) of $[Fe(pzTp)(CN)_3]^{-}$ from XRD measurements and DFT optimized geometries.

	$[Fe(pzTp)(CN)_3]^{-}$			
	XRD	doublet state	quartet state	sextet state
Fe-N1	1.979	1.983	2.008	2.178
Fe–N2	1.984	1.972	1.989	2.178
Fe–N3	1.966	2.016	2.368	2.144
Fe–C1	1.922	1.920	1.955	2.116
Fe–C2	1.915	1.923	1.954	2.115
Fe–C3	1.921	1.911	2.166	2.118
N1–Fe–N2	87.399	88.350	89.873	81.870
N1–Fe–N3	88.867	87.153	81.191	82.249
N1–Fe–C1	178.187	178.524	171.226	170.668
N1–Fe–C2	93.027	90.613	91.020	90.139

N1–Fe–C3	89.340	93.050	92.045	90.193
N2–Fe–N3	87.168	87.516	81.216	81.829
N2–Fe–C1	92.200	90.639	90.921	90.744
N2–Fe–C2	178.107	178.529	171.260	169.668
N2–Fe–C3	92.549	93.071	92.246	90.682
N3–Fe–C1	92.879	91.748	90.295	91.097
N3–Fe–C2	90.996	91.411	90.330	90.648
N3–Fe–C3	178.196	179.384	170.554	170.016
C1–Fe–C2	87.431	90.399	86.889	96.500
C1–Fe–C3	88.912	88.059	96.655	95.617
C2–Fe–C3	89.301	88.005	96.410	95.909

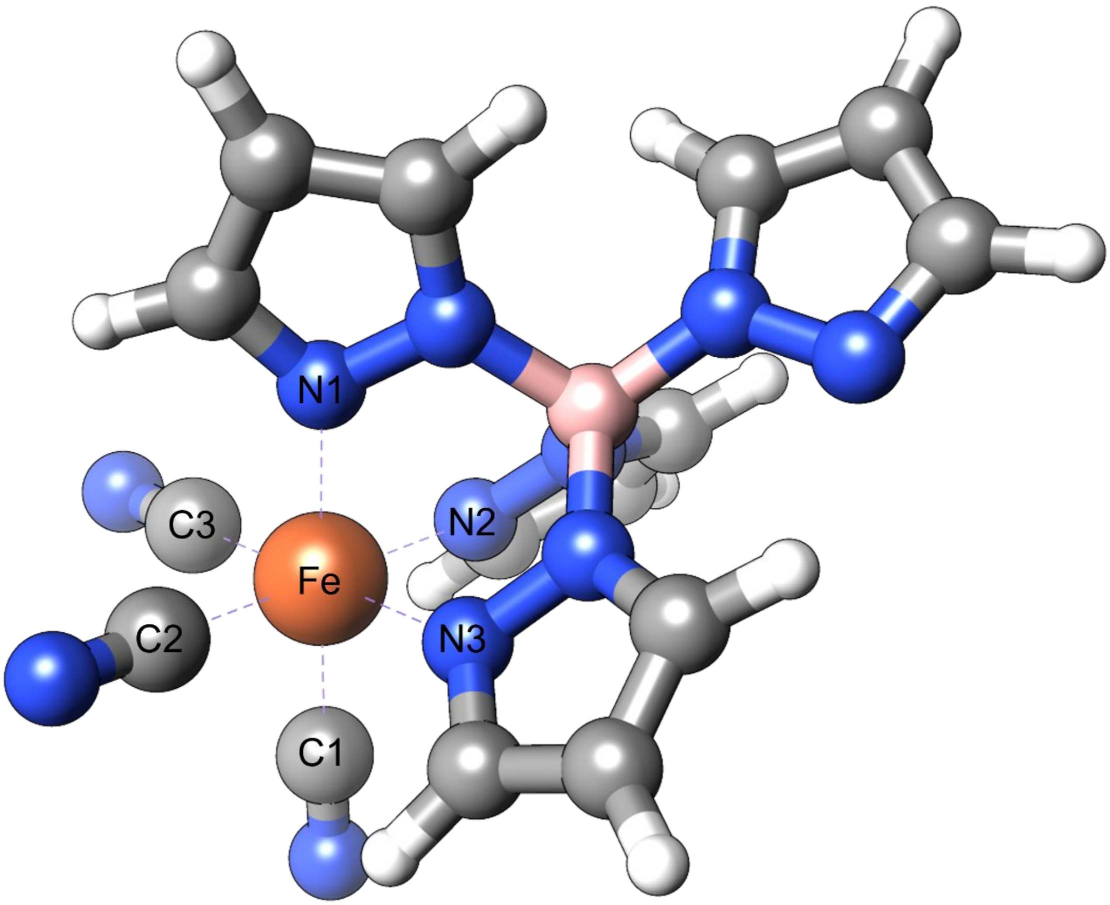

Table S2. Orbitals used in the CASSCF(5,5)/FIC-NEVPT2 calculations for $[Fe(pzTp)(CN)_3]^-$

#	E (Hartrees)	Orbital	#	E (Hartrees)	Orbital
103 (d_{yz})	-0.155856	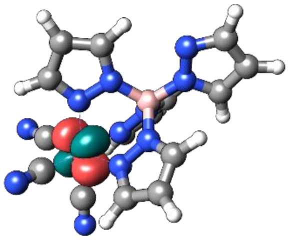	106 (d_{z^2})	-0.002125	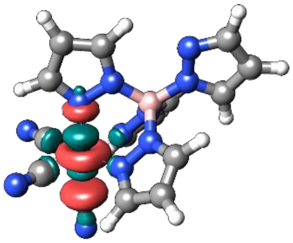
104 (d_{xz})	-0.155804	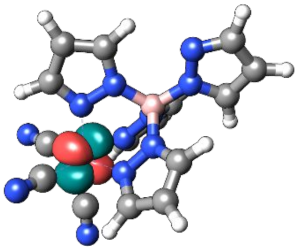	107 ($d_{x^2-y^2}$)	0.001122	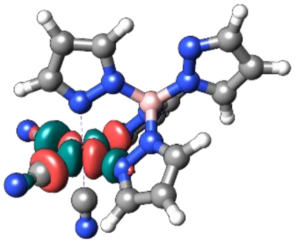
105 (d_{xy})	-0.155772	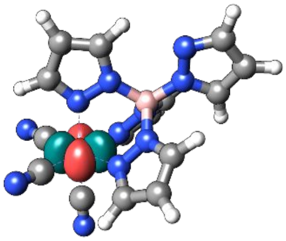			

Metal-centered excited states analysis

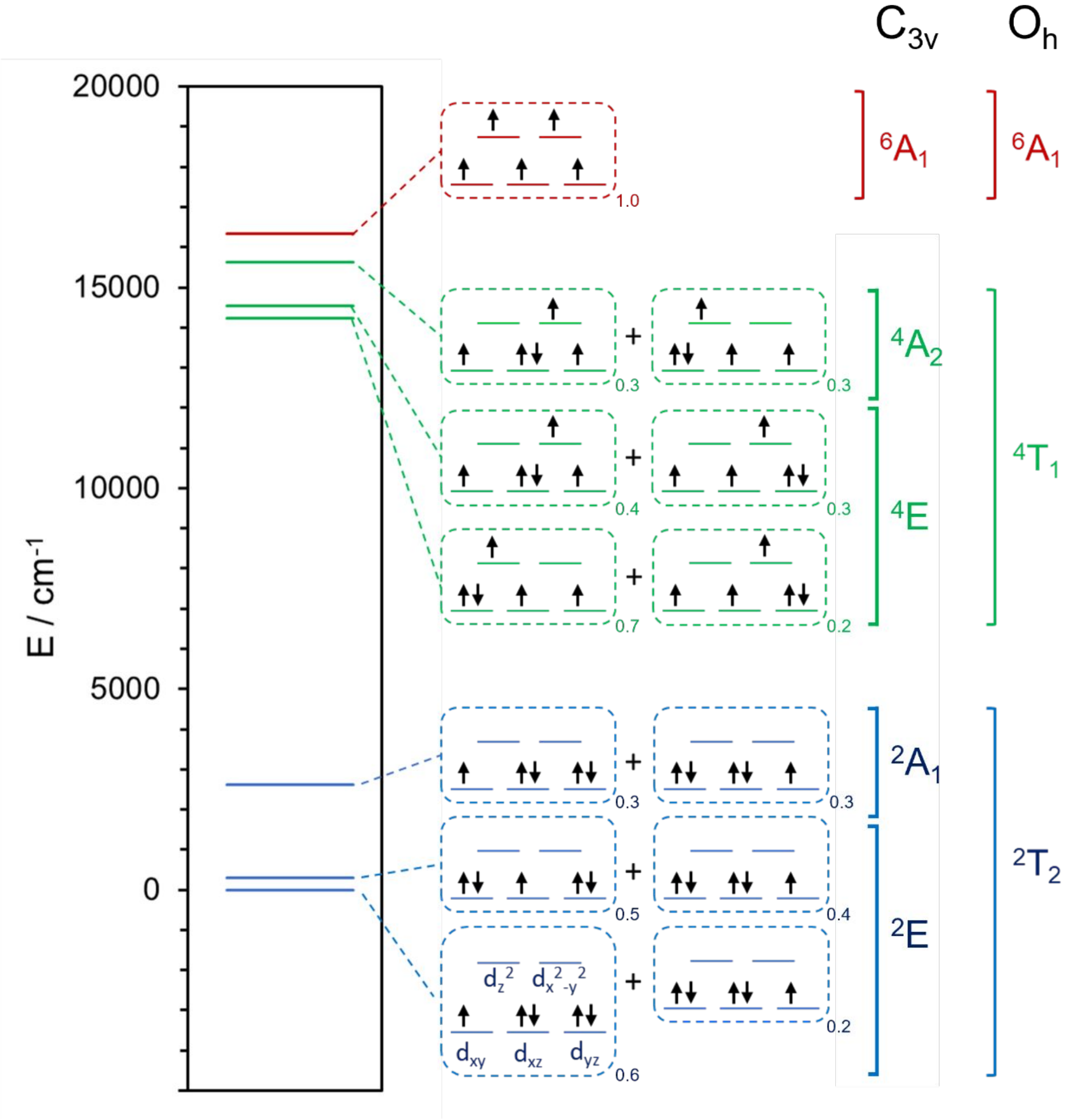

Figure S4. CASSCF(5,5)/FIC-NEVPT2 results of $[Fe(pzTp)(CN)_3]^-$. Energies in cm^{-1}. The two highest contributions for each microstate are shown, only contribution over 0.2 (20%) are shown. Note that ^2E and ^4E states are quasi-degenerated.

Table S3. Calculated 50 lowest electronic transitions for compound $[Fe(pzTp)(CN)_3]^{-}$, their energies (in nm) and oscillator strength (in cgs units).

Transition number	Wavelength / nm	Osc. Strength / cgs	Transition number	Wavelength / nm	Osc. Strength / cgs
1	6010.18211	0.000018319	26	293.066528	0.006373390
2	2751.00754	0.000099136	27	291.704499	0.003281780
3	501.277038	0.000003618	28	290.238024	0.003232750
4	495.650569	0.000016789	29	283.852341	0.003366250
5	466.837934	0.000267392	30	282.80612	0.008744250
6	465.546008	0.003593200	31	276.950474	0.000019111
7	441.799322	0.000074204	32	275.591901	0.000083578
8	425.517575	0.000278607	33	273.293441	0.001996310
9	421.988175	0.024203500	34	271.517929	0.001116640
10	410.984653	0.052916600	35	266.090037	0.000084526
11	395.952165	0.000698521	36	263.377834	0.016390300
12	393.503403	0.009234470	37	258.999037	0.000930363
13	381.379884	0.001580250	38	258.999037	0.000197749
14	379.573959	0.001157280	39	257.748508	0.006726140
15	376.388277	0.005883280	40	256.185694	0.030252300
16	371.718816	0.001092730	41	255.873178	0.000263495
17	364.509086	0.008147320	42	253.894266	0.016762000
18	361.013179	0.000245064	43	251.707767	0.005687720
19	356.566612	0.000016313	44	250.354598	0.001002940
20	333.536505	0.030786200	45	249.938297	0.000973591
21	323.421157	0.004584600	46	247.233007	0.001090790
22	318.369508	0.000332624	47	246.920932	0.011739400
23	311.745302	0.000105613	48	245.464788	0.003229080
24	307.22798	0.001153210	49	244.632857	0.001255610
25	305.442947	0.002706960	50	242.241663	0.004648000

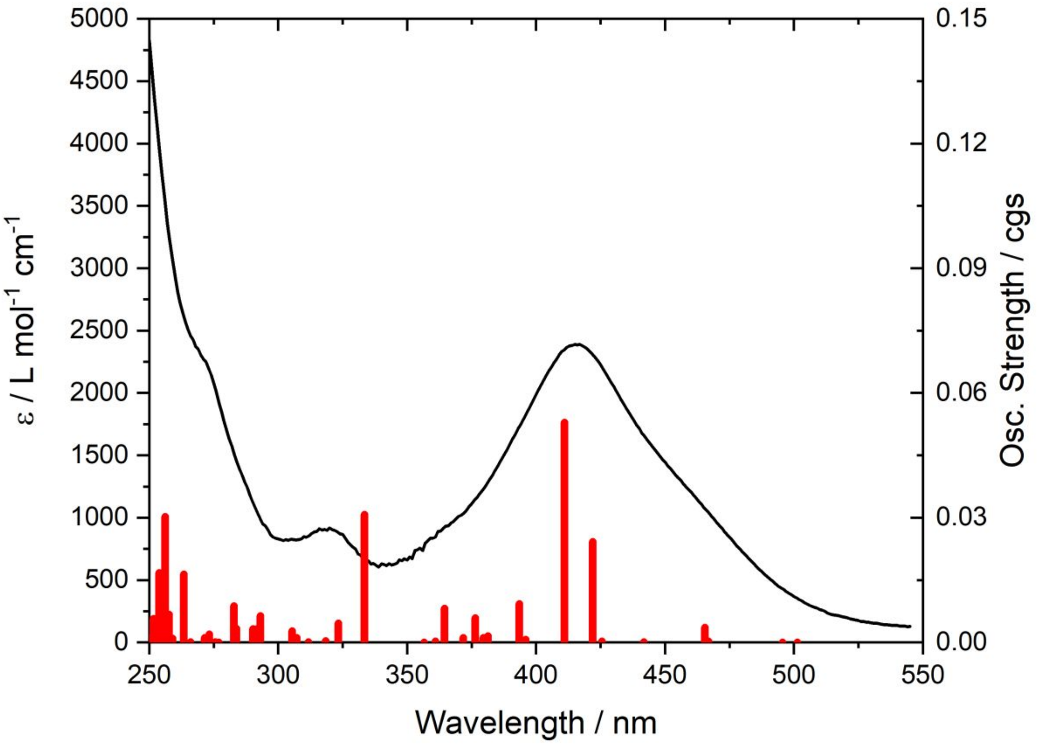

Figure S5. Experimental UV-Vis spectrum of compound $[Fe(pzTp)(CN)_3]^-$ in chloroform and calculated oscillator strength of the calculated electronic transitions.

Electron density difference maps for [Fe(pzTp)(CN)$_3$]$^-$ (Blue: density loss; Purple: density gain).

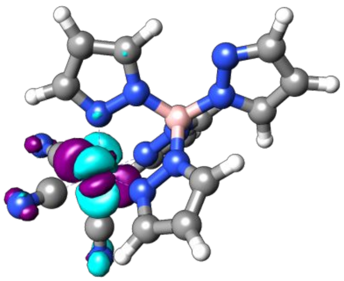

#1 - 6010.18 nm – MC

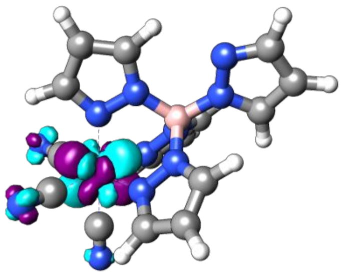

#2 - 2751.01 nm – MC

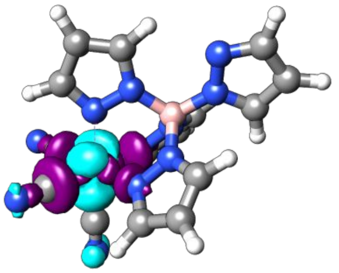

#3 - 501.28 nm – MLCT / MC

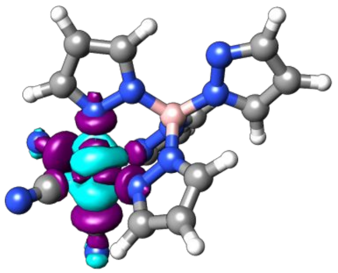

#4 - 495.65 nm – MLCT / MC

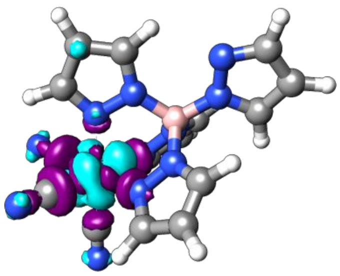

#5 - 466.84 nm – MLCT / MC

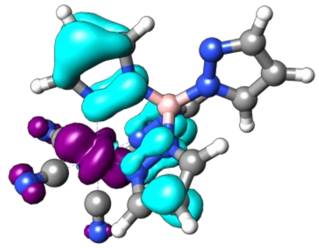

#6 - 465.55 nm – LMCT

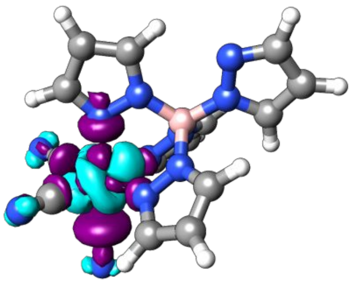

#7 - 441.80 nm – MLCT / MC

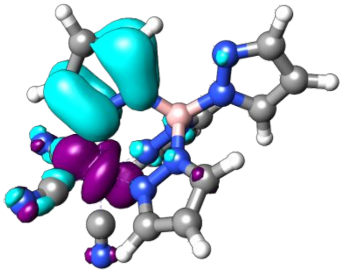

#8 - 425.52 nm - LMCT

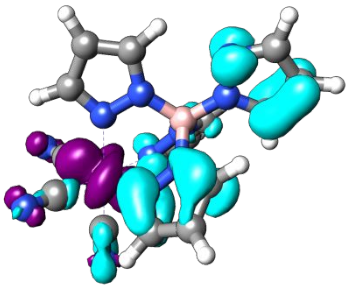

#9 - 421.99 nm – LMCT

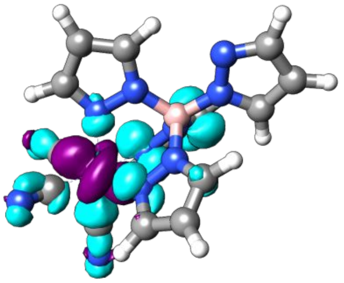

#10 - 410.98 nm – LMCT

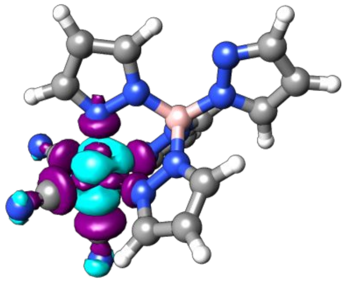

#11 - 395.95 nm – MLCT / MC

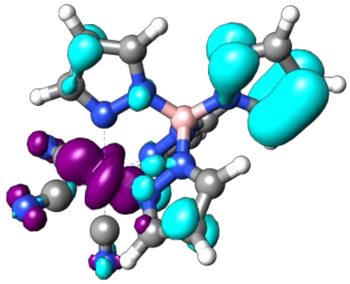

#12 - 393.50 nm - LMCT

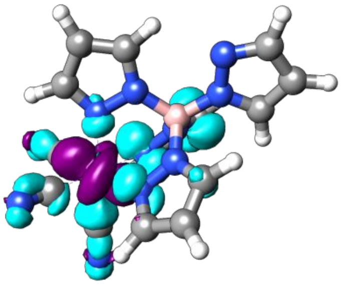

#10 - 410.98 nm – LMCT

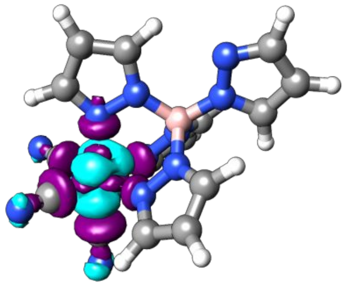

#11 - 395.95 nm – MLCT / MC

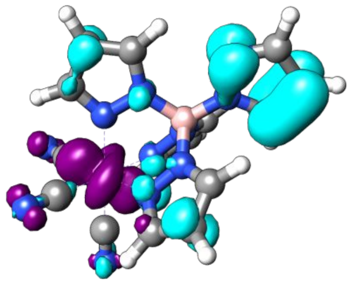

#12 - 393.50 nm - LMCT

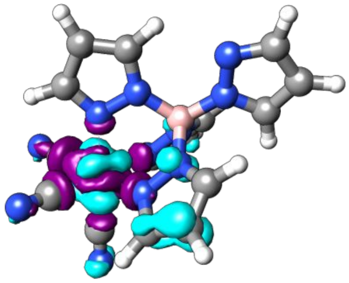

#13 - 381.38 nm – LMCT / MC / MLCT / LLCT

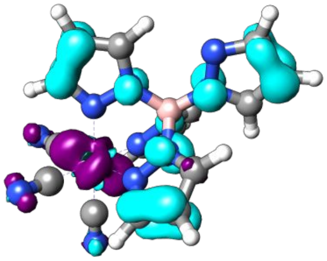

#14 - 379.57 nm - LMCT

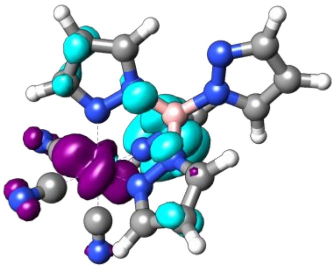

#15 - 376.39 nm – LMCT

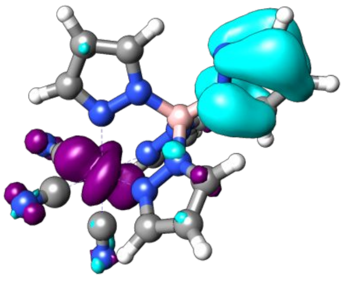

#16 - 371.72 nm - LMCT

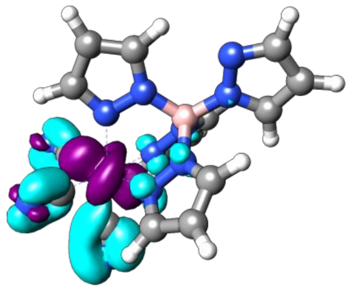

#17 - 364.51 nm - LMCT

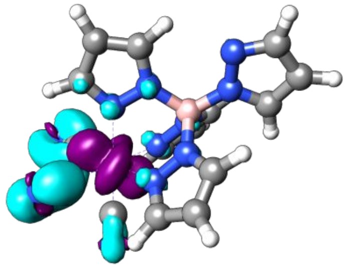

#18 - 361.01 nm – LMCT

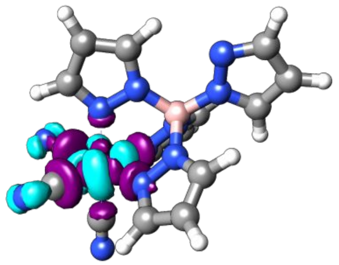

#19 - 356.57 nm – MLCT / MC

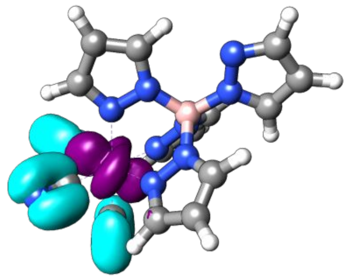

#20 - 333.54 nm - LMCT

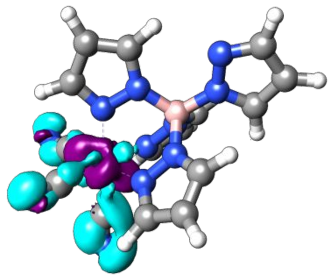

#21 - 323.42 nm - LMCT

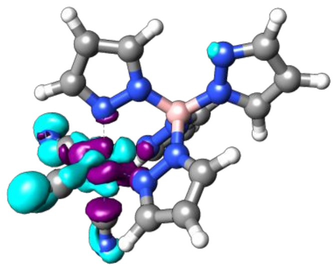

#22 - 318.37 nm – LMCT / MC

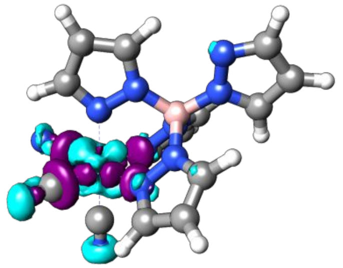

#23 - 311.75 nm – MLCT / MC

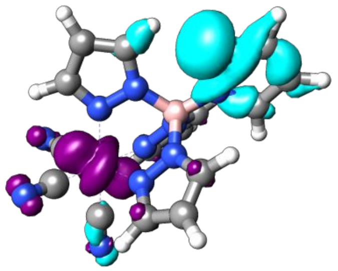

#24 - 307.23 nm - LMCT

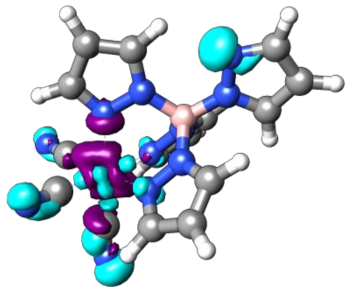

#25 - 305.44 nm – LMCT / MC

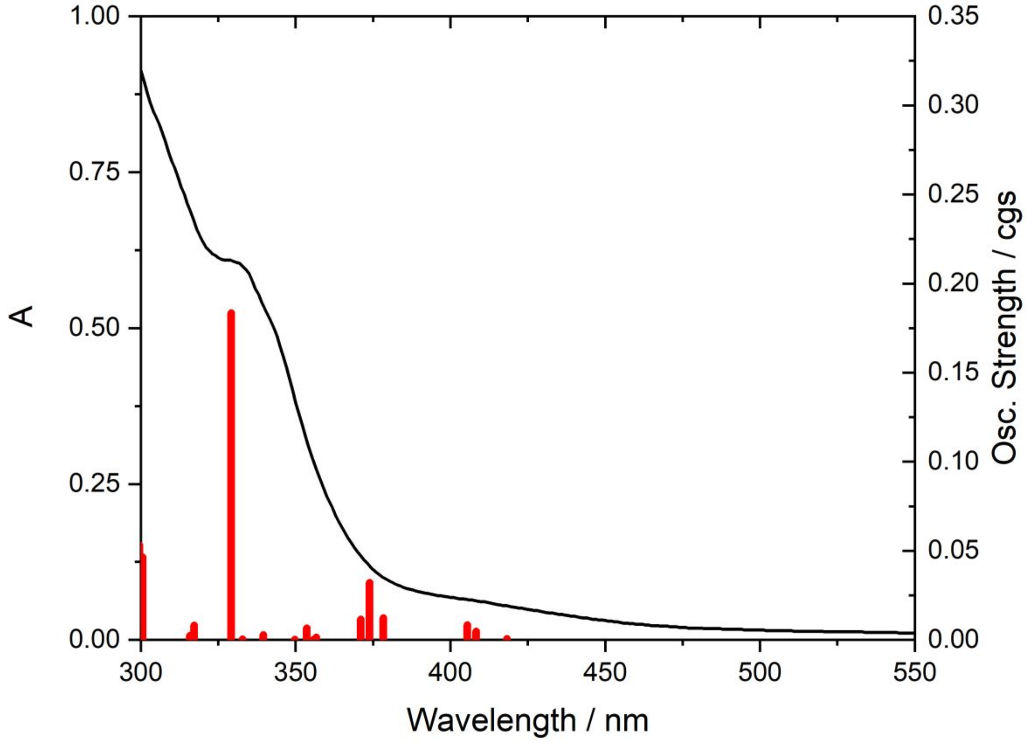

Figure S6. Experimental UV-Vis spectrum of compound $[Fe(pzTp)(CN)_3]^{2-}$ in chloroform (black) and oscillator strength of the calculated electronic transitions (red).

Electron density difference maps for [Fe(pzTp)(CN)$_3$]$^{2-}$ (Blue: density loss; Purple: density gain).

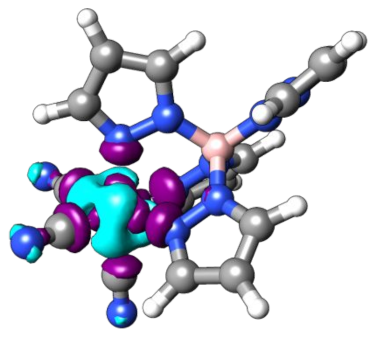

#1 – 418.30 nm – MC

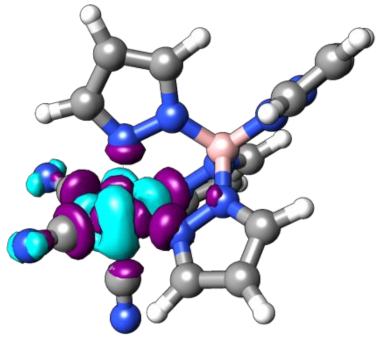

#2 – 408.30 nm – MC

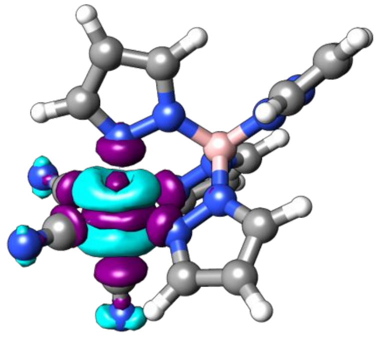

#3 – 405.50 nm – MC

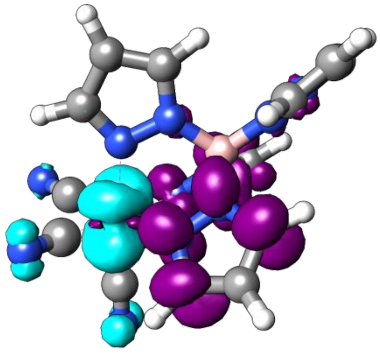

#4 – 378.40 nm – MLCT

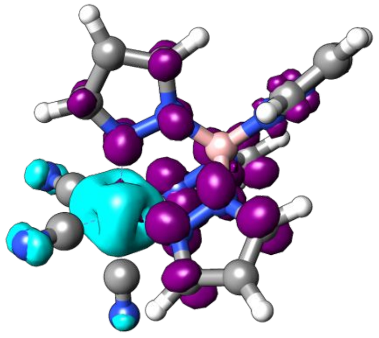

#5 – 373.90 nm – MLCT

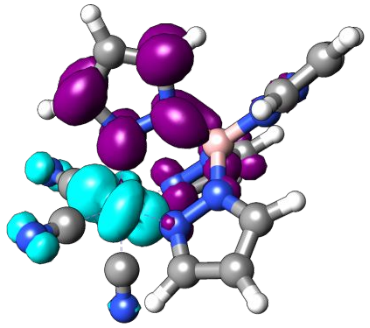

#6 – 371.00 nm – MLCT

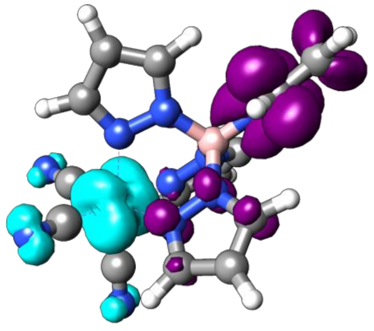

#7 – 356.70 nm – MLCT

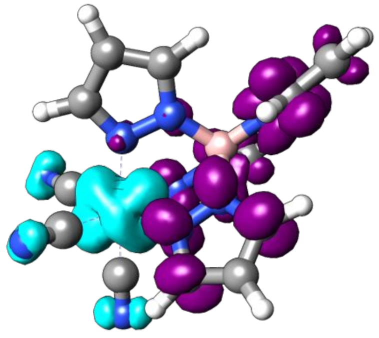

#8 – 355.80 nm – MLCT

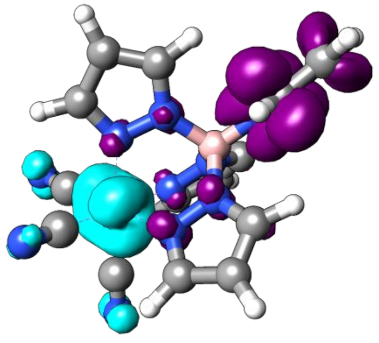

#9 – 353.60 nm – MLCT

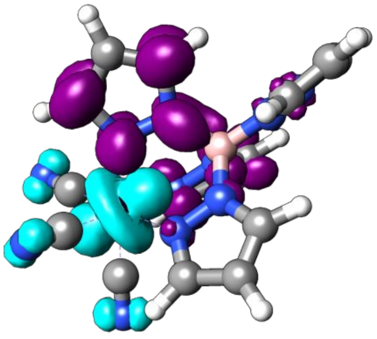
#10 – 349.70 nm – MLCT

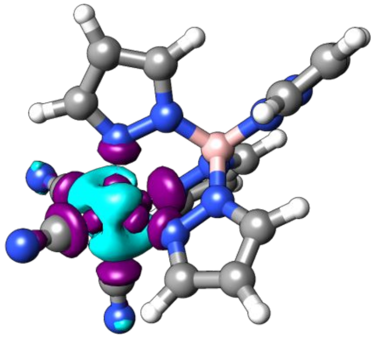
#11 – 339.60 nm – MC

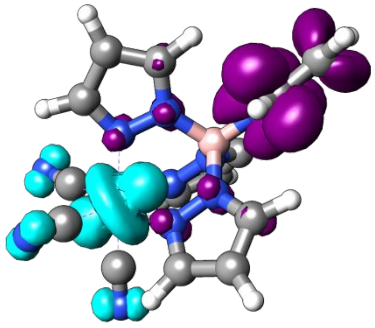
#12 – 332.90 nm – MLCT

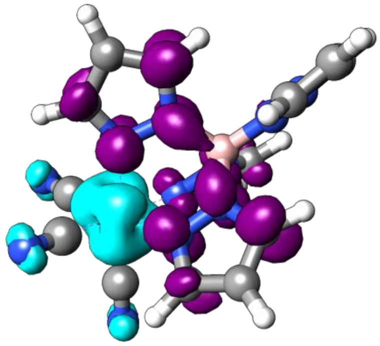
#13 – 329.20 nm – MC

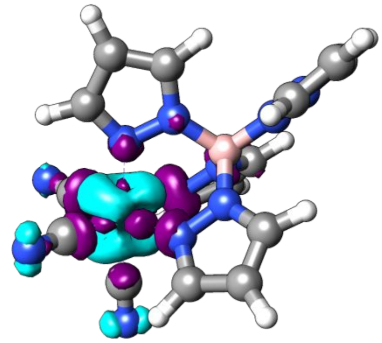
#14 – 317.20 nm – MC

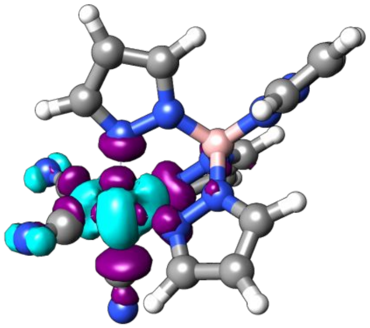
#15 – 315.90 nm – MC

Table S4. Calculated 50 lowest electronic transitions for compound $[Fe(pzTp)(CN)_3]^{2-}$, their energies (in nm) and oscillator strength (in cgs units).

Transition number	Wavelength / nm	Osc. Strength / cgs	Transition number	Wavelength / nm	Osc. Strength / cgs
1	418.30	0.000602542	26	245.90	0.000057341
2	408.30	0.004814050	27	243.50	0.002585520
3	405.50	0.008418160	28	243.30	0.003475840
4	378.40	0.012400400	29	242.70	0.009034270
5	373.90	0.032115800	30	235.30	0.002561950
6	371.00	0.011541000	31	235.20	0.002537030
7	356.70	0.001389300	32	233.80	0.000030700
8	355.80	0.000336363	33	231.70	0.000293691
9	353.60	0.006601040	34	231.50	0.001366200
10	349.70	0.000227290	35	227.90	0.005868290
11	339.60	0.002890470	36	226.90	0.003403390
12	332.90	0.000375454	37	226.90	0.002397090
13	329.20	0.183425000	38	226.60	0.000382538
14	317.20	0.008110920	39	221.80	0.001672470
15	315.90	0.002450060	40	220.30	0.002174040
16	300.60	0.046313400	41	220.20	0.000530048
17	299.60	0.053227100	42	219.70	0.011260500
18	289.00	0.000019479	43	219.20	0.006980160
19	261.50	0.007499170	44	218.60	0.028209900
20	261.30	0.001694150	45	217.30	0.000969374
21	256.30	0.003533450	46	217.20	0.005285090
22	255.70	0.008345870	47	215.90	0.024449000
23	254.20	0.004134960	48	215.80	0.013089900
24	253.60	0.008034070	49	214.00	0.005171790
25	250.60	0.000075598	50	213.60	0.006729620

4. Transient absorption and time-resolved measurements

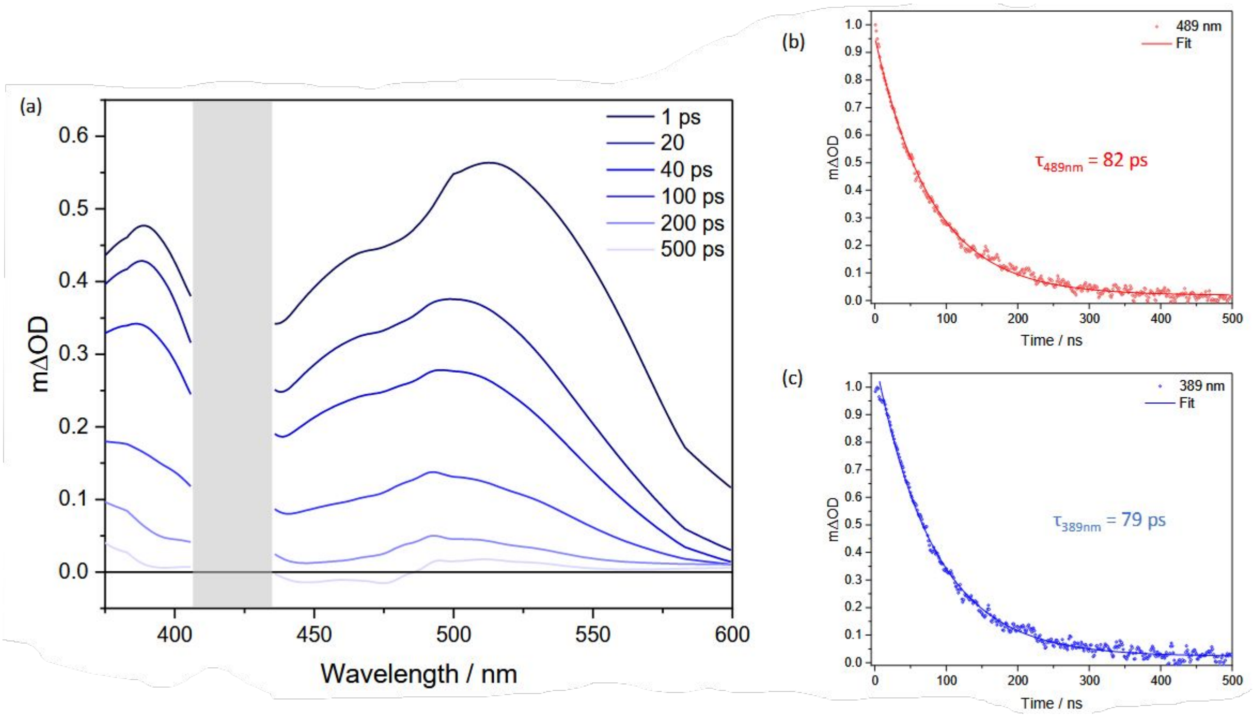

Figure S7. Femtosecond transient absorption spectra of 20 μM $[Fe(pzTP)(CN)_3]^-$ (a) in deaerated $CHCl_3$ at 20 °C, obtained after excitation at 420 nm (a) at different time delays. Decays of the ESA signal with monoexponential fits at 489 nm (b) and 389 nm (c).

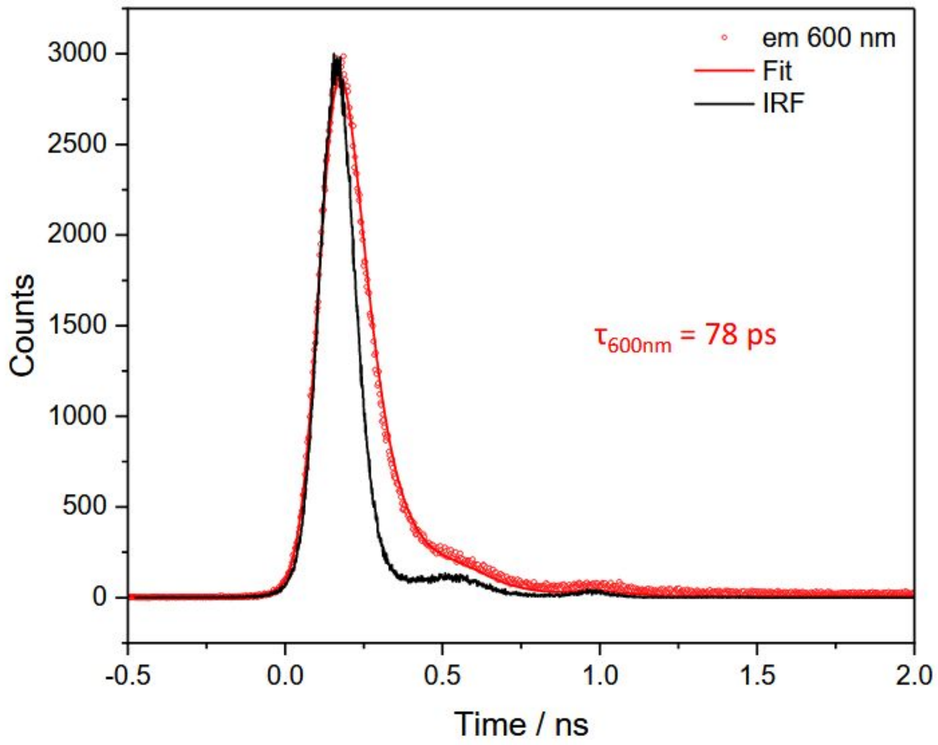

Figure S8. Decay of $[Fe(pzTP)(CN)_3]^-$ emission at 600 nm after 405 nm excitation of a 15 μM $[Fe(pzTp)(CN)_3]^-$ solution in argon-saturated $CHCl_3$ at 20 °C (red circles) obtained with time-correlated single photon counting (TCSPC) technique. Reconvolution fit of the emission data (red trace). Instrument response function (black trace).

5. Absorption spectra

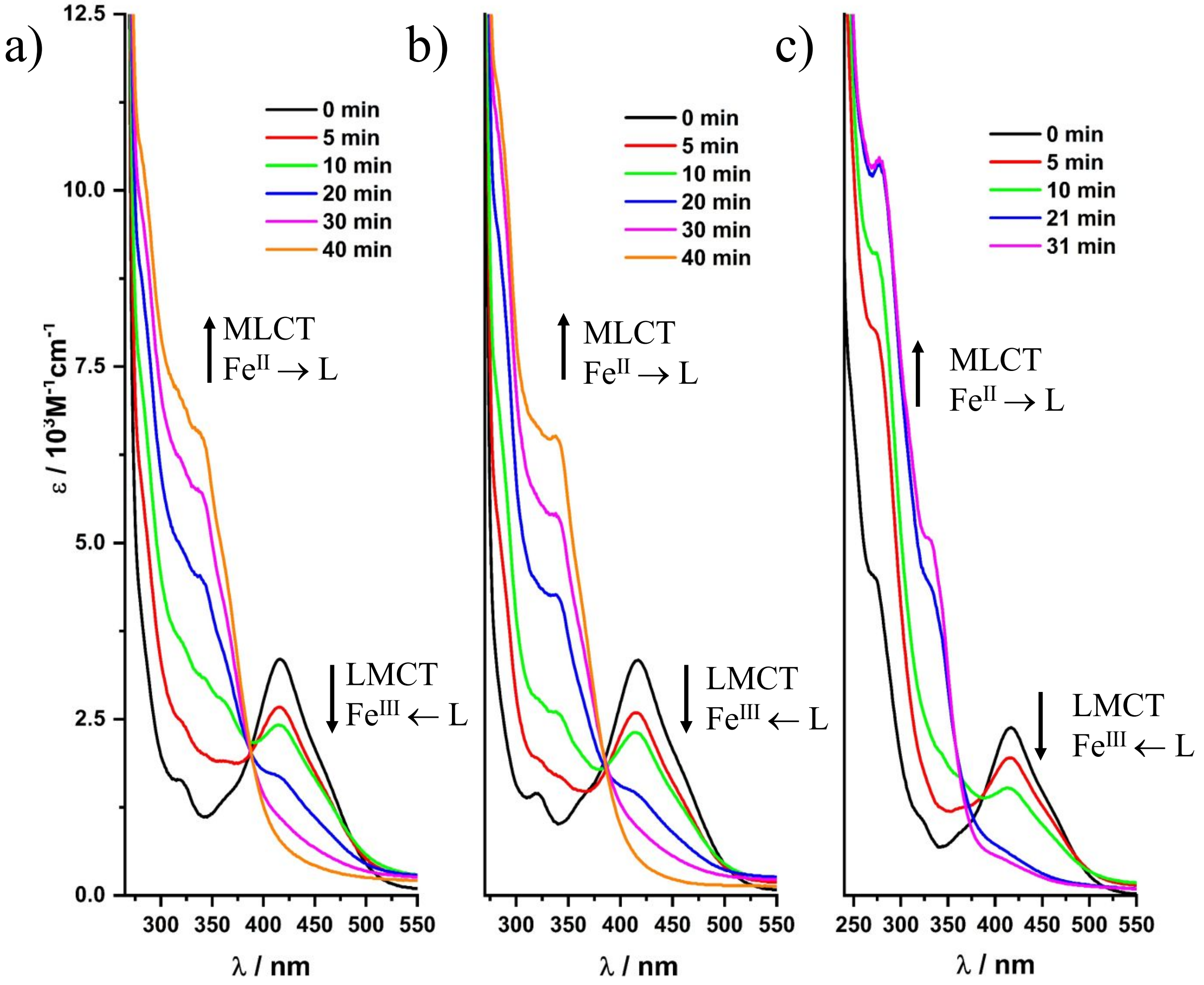

Figure S9. UV-Vis spectra of $[Fe(pzTp)(CN)_3]^-$ (0.1 mM) in $CDCl_3$ at different photo-reactions times and in the presence of different electron donors (15 mM): a) isopropanol, b) benzyl alcohol and c) phenylethanol.

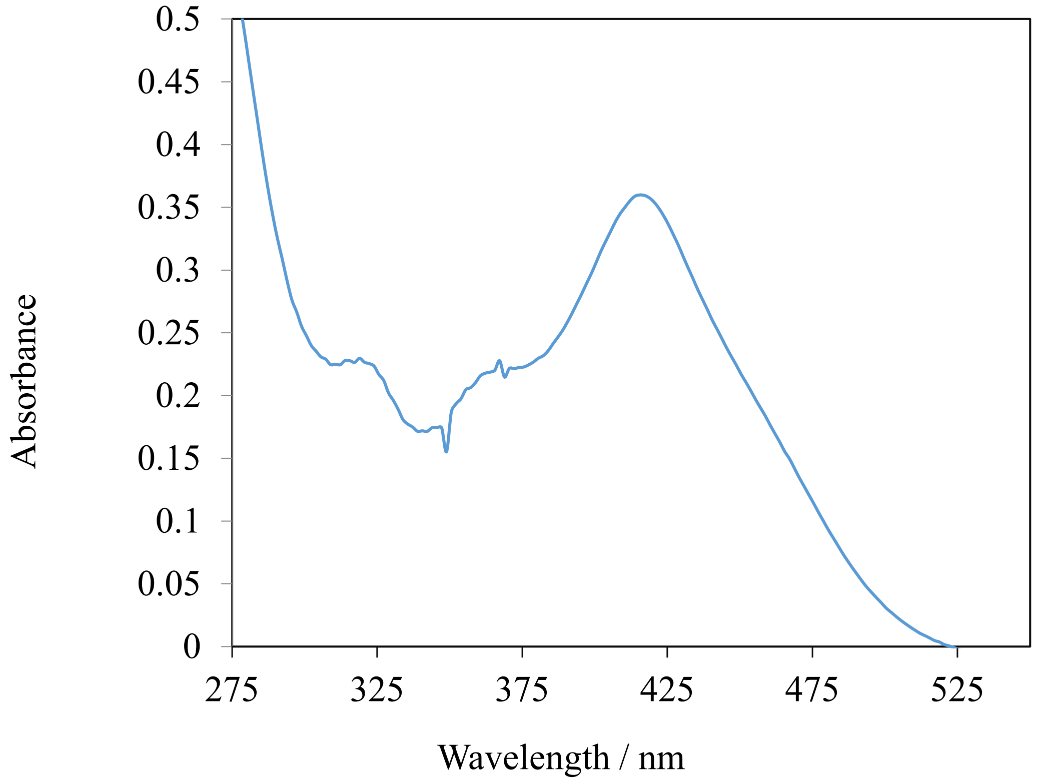

Figure S10. UV-Vis spectra of $[Fe(pzTp)(CN)_3]^-$ (0.1 mM) in $CDCl_3$ after 20 min upon irradiation at 440 nm.

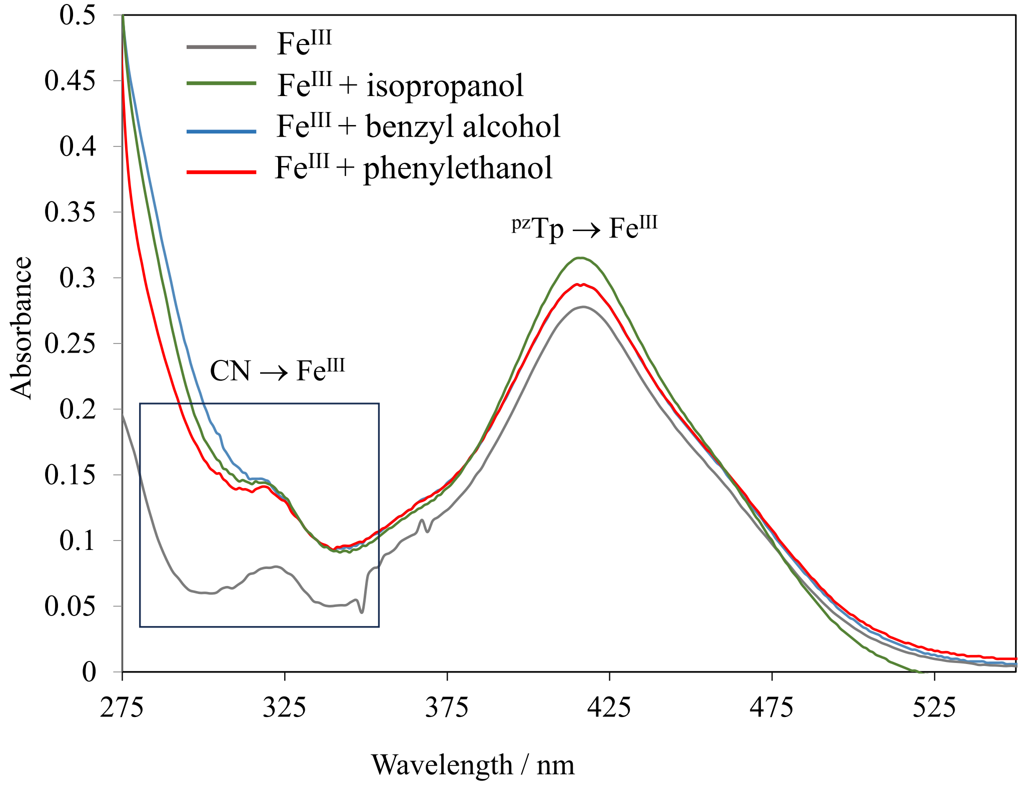

Figure S11. UV-Vis spectra of $[Fe(pzTp)(CN)_3]^-$ (0.1 mM) in $CDCl_3$ (gray) and in the presence of different substrates before any photochemical reaction.

6. FTIR spectra

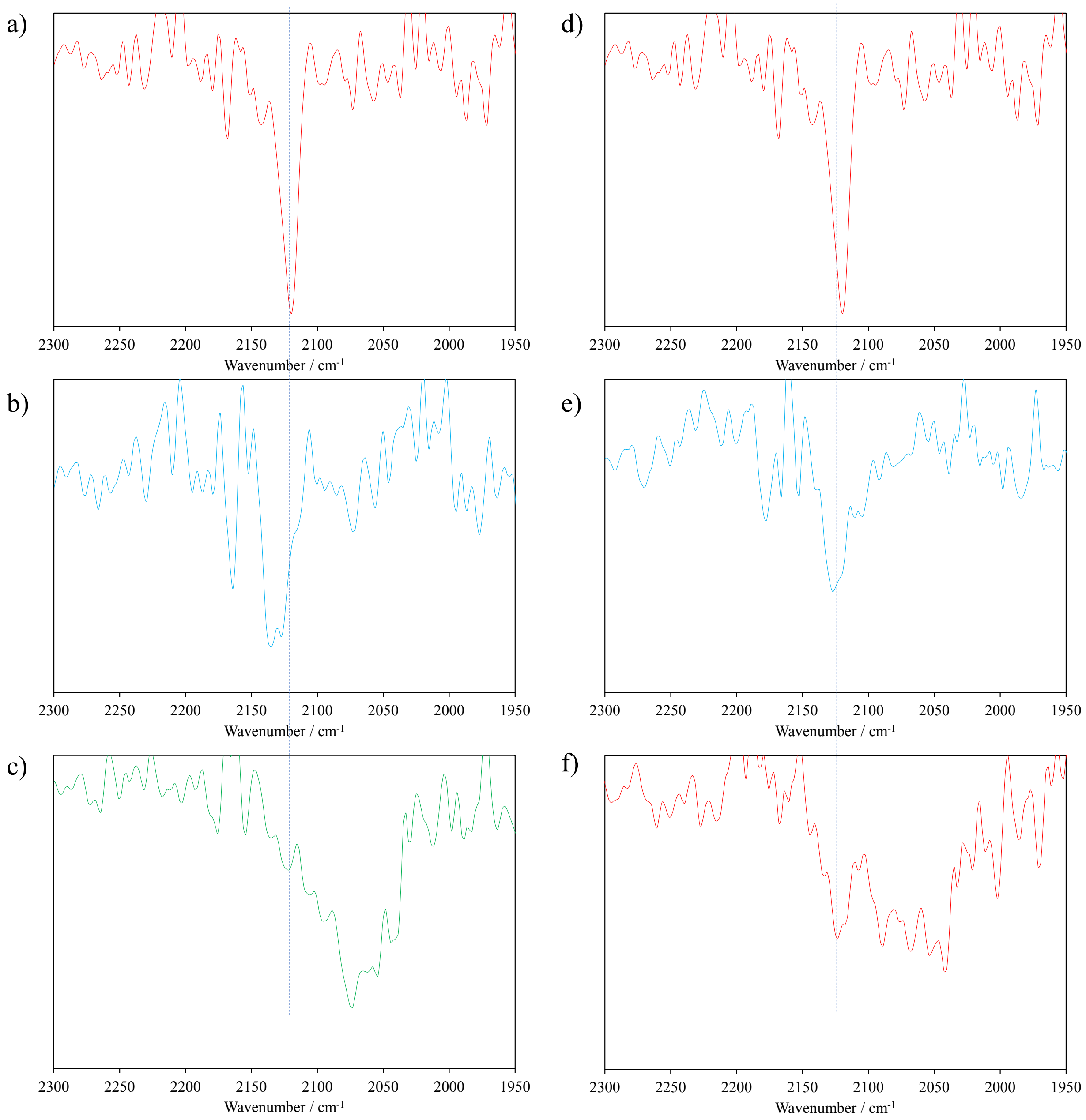

Figure S12. FTIR spectra highlight the cyanide stretching region for: a) and d) the $[Fe(pzTp)(CN)_3]^-$, b) $[Fe(pzTp)(CN)_3]^-$ + benzyl alcohol (before irradiation), c) $[Fe(pzTp)(CN)_3]^-$ + benzyl alcohol (after continuous irradiation at 440 nm during 30 min), e) $[Fe(pzTp)(CN)_3]^-$ + phenylethanol (before irradiation) and f) $[Fe(pzTp)(CN)_3]^-$ + phenylethanol (after continuous irradiation at 440 nm during 30 min).

7. Photochemical studies

General set up for a typical photochemical experiment: in 1 ml quartz cuvette (1 cm optical path), 1 ml of a $CDCl_3$ solution containing 5 mM of the Fe complex was placed and subsequently 4 equivalents of the alcohol substrate were added. The cuvette was closed gas-tight with a septum and the solution was degassed for 5 minutes. The solution was irradiated under stirring with a 440 nm LED lamp during the specified time and a UV-vis spectrum was obtained (Figure S9). For the quantification of the product via ^{1}H-NMR, a similar procedure using 4 ml gas-tight vials was employed; a known amount of dioxane was added as internal standard at the end of the reaction and the solution was placed in an NMR tube for its analysis as crude reaction. Integral of the product signal was performed relative to the integral of the dioxane peak (approximately 3.8 ppm), which allowed for NMR yield quantification. The peaks of the product were compared to the NMR of the pure products obtained from commercial sources and to literature values to confirm its nature.

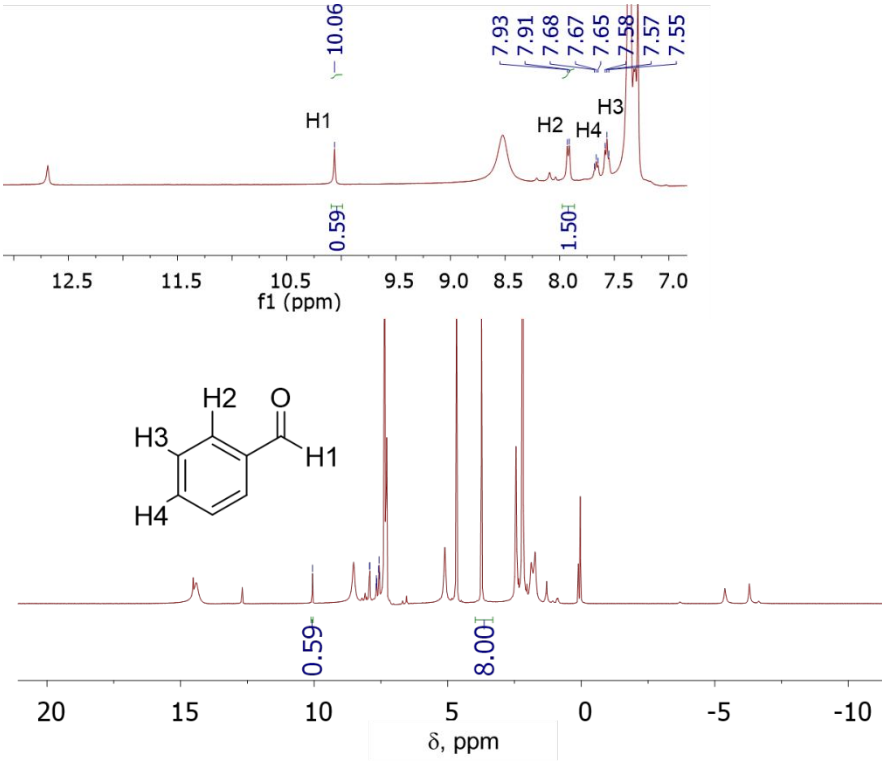

Figure S13. ^{1}H-NMR spectra of the crude of the photochemical reaction between the ^{2}LMCTexcited state of $[Fe(pzTp)(CN)_3]^-$ ([Fe^{III}], 5mM) and benzyl alcohol (20 mM) in $CDCl_3$ upon irradiation with a blue LED (440 nm) during 3 h.. The top spectrum shows a zoom of the aromatic region.

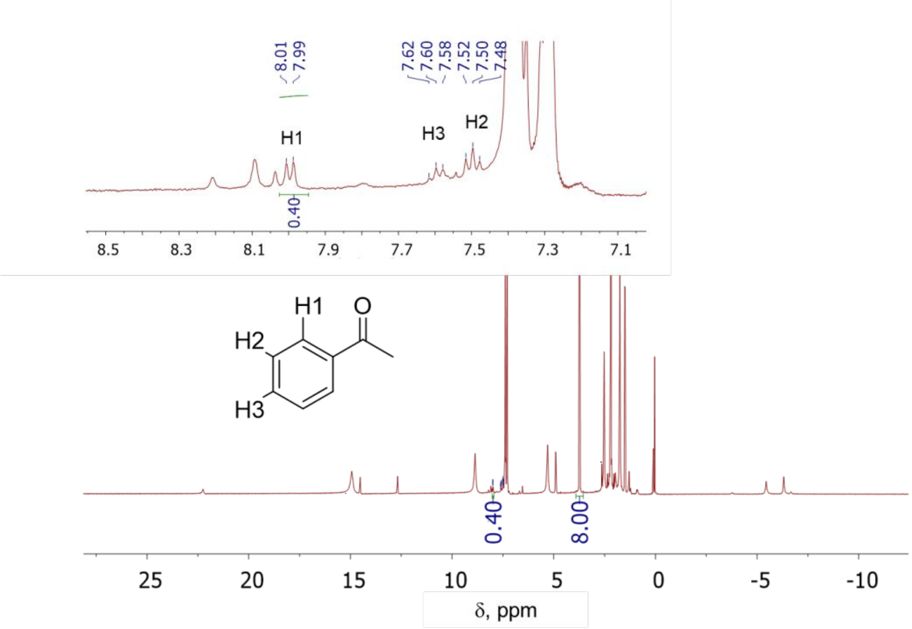

Figure S14. ^{1}H-NMR spectra of the crude of the photochemical reaction between the [2]LMCTexcited state of $[Fe(pzTp)(CN)_3]^{-}$ ($[Fe^{III}]$, 5mM) and 1-phenylethanol (20 mM) in $CDCl_3$ upon irradiation with a blue LED (440 nm) during 3 h. The top spectrum shows a zoom of the aromatic region.

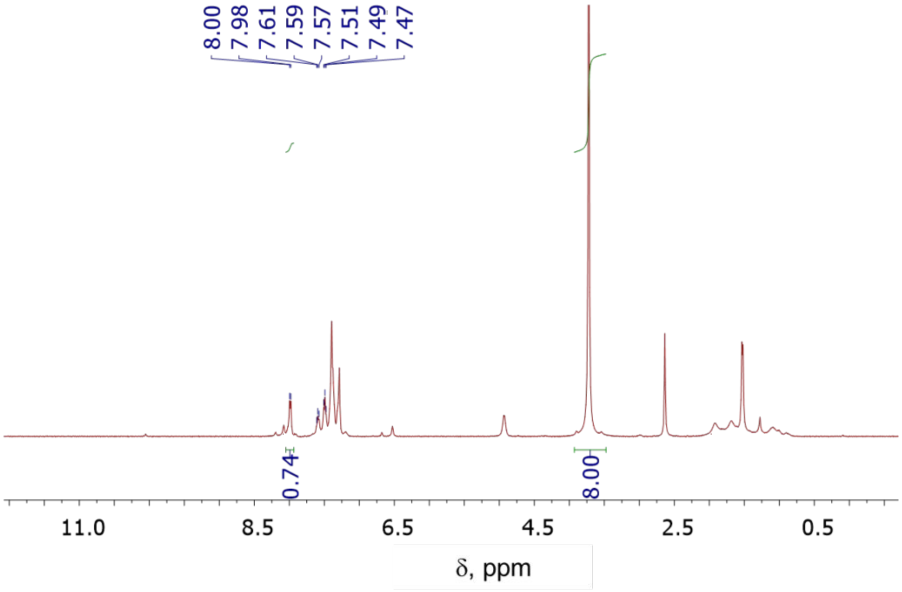

Figure S15. ^{1}H-NMR spectra of the crude of the photochemical reaction between the [2]LMCT excited state of $[Fe(pzTp)(CN)_3]^{-}$ ($[Fe^{III}]$, 5mM) and 1-phenylethanol (20 mM) in $CDCl_3$ upon irradiation with a blue LED (440 nm) during 6 h.

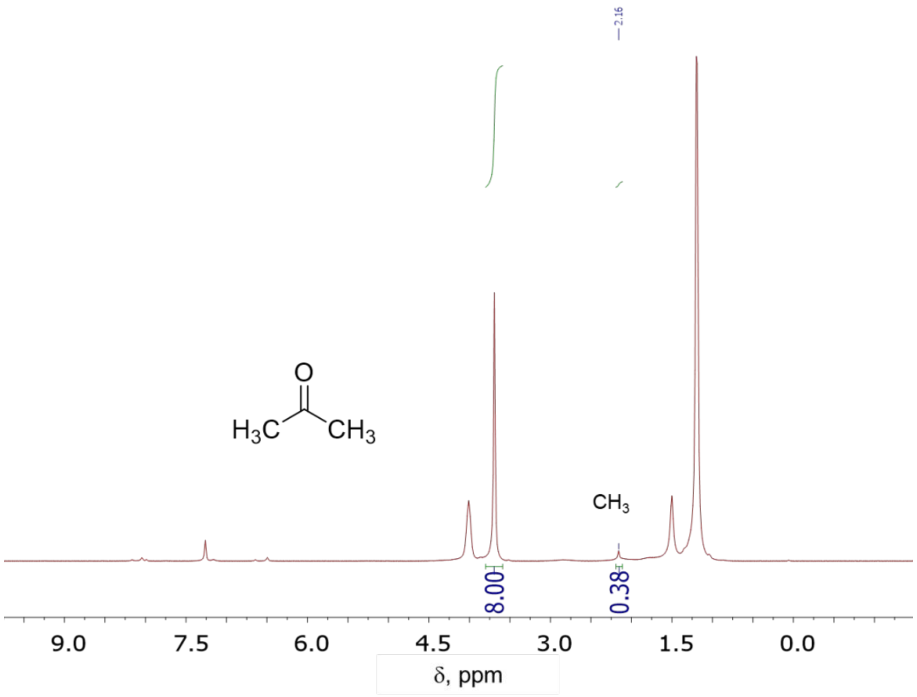

Figure S16. ^{1}H-NMR spectra of the crude of the photochemical reaction between the ^{2}LMCTexcited state of $[Fe(pzTp)(CN)_3]^{-}$ ($[Fe^{III}]$, 5mM) and isopropanol (50 mM) in $CDCl_3$ upon irradiation with a blue LED (440 nm) during 6 h.

Control reactions:

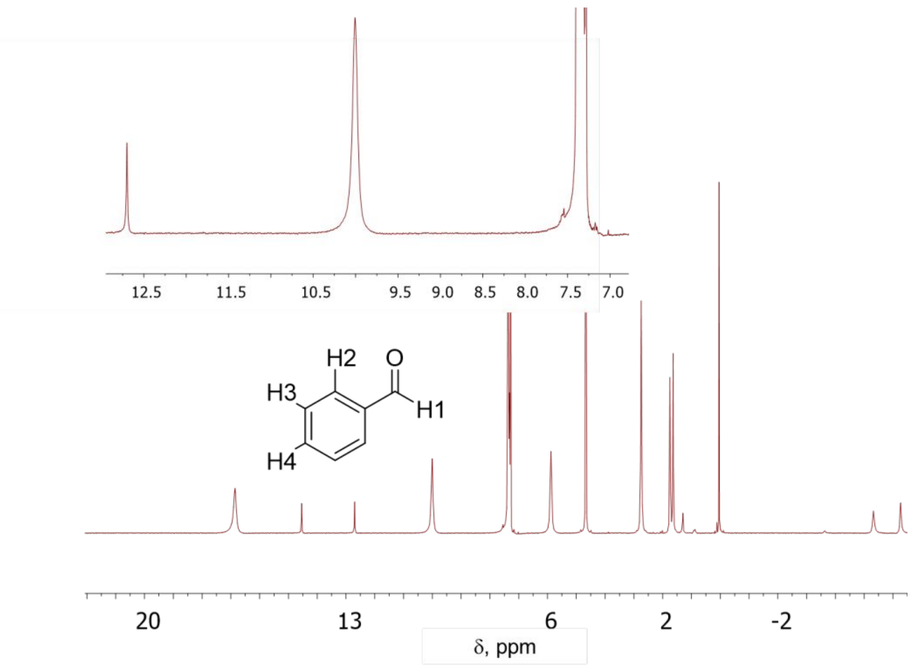

Figure S17. ^{1}H-NMR spectra of the crude of the control reaction between the $[Fe(pzTp)(CN)_3]^-$ ($[Fe^{III}]$, 5mM) and benzyl alcohol (20 mM) in $CDCl_3$ without irradiation. The top spectrum shows a zoom of the aromatic region.

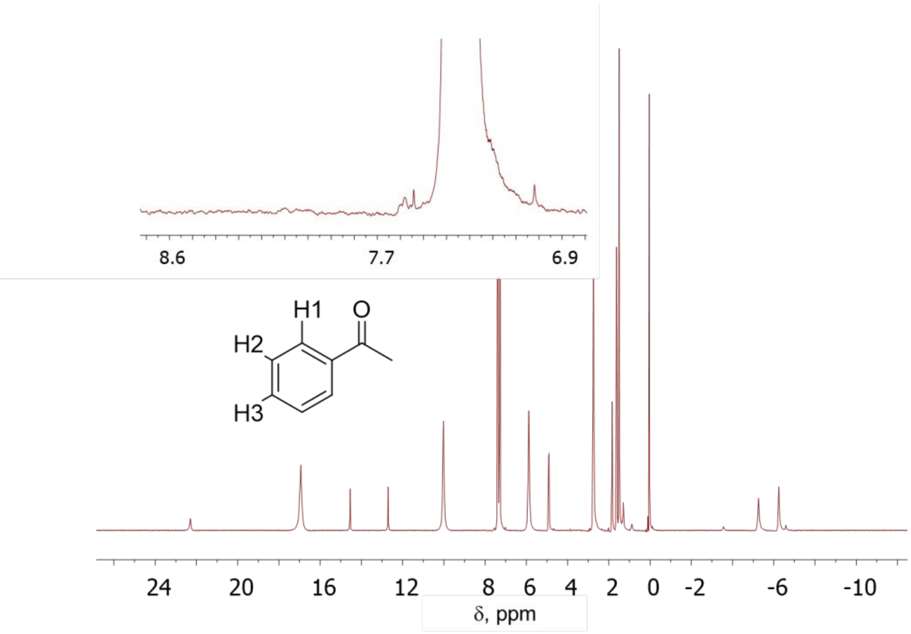

Figure S18. ^{1}H-NMR spectra of the crude of the control reaction between the $[Fe(pzTp)(CN)_3]^-$ ($[Fe^{III}]$, 5mM) and 1-phenylethanol (20 mM) in $CDCl_3$ without irradiation.. The top spectrum shows a zoom of the aromatic region.

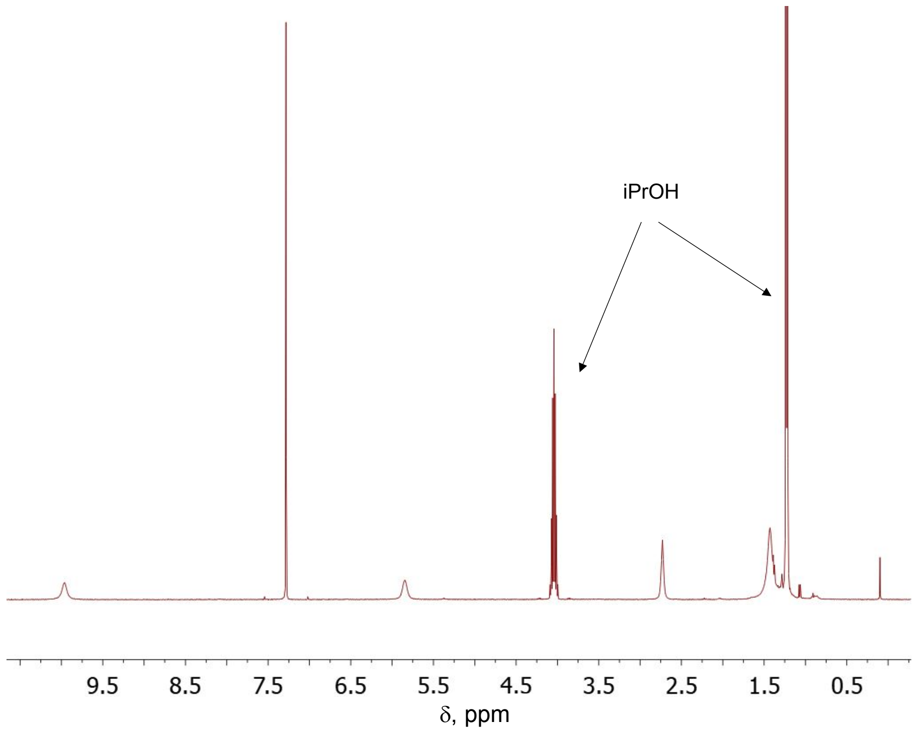

Figure S19. ^{1}H-NMR spectra of the crude of the control reaction between the $[Fe(pzTp)(CN)_3]^-$ ($[Fe^{III}]$, 5mM) and isopropanol (50 mM) in $CDCl_3$ without irradiation. The top spectrum shows a zoom of the aromatic region.

Stern-Volmer

Stern-Volmer plots were employed to interrogate the kinetics of the photo-oxidation processes by monitoring the emission intensity (I) versus the concentration of the quencher (alcohol substrate). The Stern-Volmer relationship follows Equation 1, where I_0 is the emission intensity in the absence of quencher and K_{SV} is the Stern-Volmer quenching. In the case of dynamic quenching, this K_{SV} is the product of the kinetic rate of the quenching process (electron transfer in this case, k_{ET}) and the lifetime of the excited state (τ_0). In the case of static quenching, K_{SV} is the equilibrium constant of the adduct formation, in our particular case, the equilibrium constant of hydrogen bonding K_{HB}. The resulting plot was fitted to a linear regression, from which K_{SV} can be extracted using the slope.

$$\frac{I_0}{I} = 1 + K_{SV}[Q] \quad \text{Eq. 1}$$

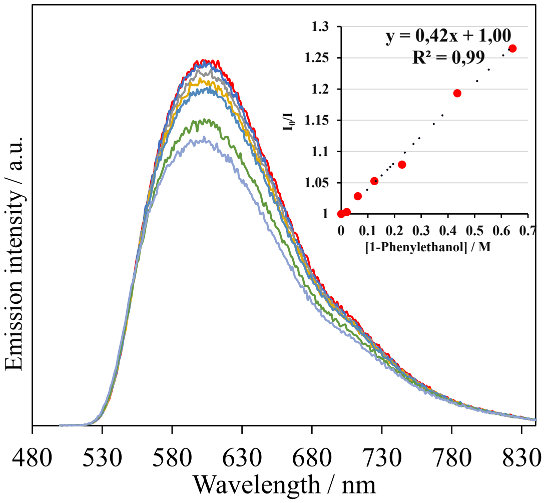

Figure S20. Emission quenching of $[Fe(pzTp)(CN)_3]^-$ monitored by steady-state emission spectra for increasing concentrations of 1-phenylethanol. Stern-Volmer plots for steady state intensity from quenching experiments (inset). A value for k_{ET} of $5.3\cdot10^9$ $M^{-1}\cdot s^{-1}$ is estimated assuming dynamic quenching.

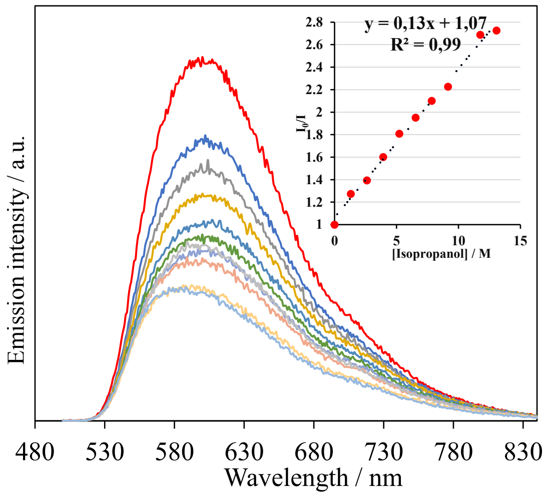

Figure S21. Emission quenching of $[Fe(pzTp)(CN)_3]^-$ monitored by steady-state emission spectra for increasing concentrations of isopropanol. Stern-Volmer plots for steady state intensity from quenching experiments (inset). A value for k_{ET} of $1.7 \cdot 10^9$ $M^{-1} \cdot s^{-1}$ is estimated assuming dynamic quenching.

Marcus analysis: For electron transfer reactions, Marcus theory correlates the kinetic rate of the process, *k*, with the overall driving force, $-\Delta G^o$, and the reorganization energy, λ, according to Equations 3-4.[3] In these equations, α is defined as the Brønsted value, which in the case of the low driving force regime, it is predicted to be a constant value of 0.5. Plotting $Ln(k_{ET})$ versus $-\Delta G^o$ allows to extract the value for α from the slope (αRT).

$$k = \kappa \nu \exp\left(-\frac{\Delta G^{\ddagger}}{RT}\right), \qquad Eq.2$$

$$\Delta G^{\ddagger} = \frac{(\lambda + \Delta G^{o})^2}{4\lambda}, \qquad Eq.3$$

$$\alpha = \frac{\delta \Delta G^{\ddagger}}{\delta \Delta G^{o}} = \frac{1}{2} + \frac{\Delta G^{o}}{2\lambda}, \qquad Eq.4$$

Using the lifetime value of the excited state (τ_0) allows us to calculate k_{ET}. Thus, we plotted $Ln(k_{ET})$ vs $-\Delta G^o$ to perform the Marcus analysis, employing the redox potential for the oxidation of each of the substrate experimentally measured via cyclic voltammetry. The linear fit between $Ln(k_{ET})$ and $-\Delta G^o$ provides a slope of 0.24 that results in a α value of 0.14.

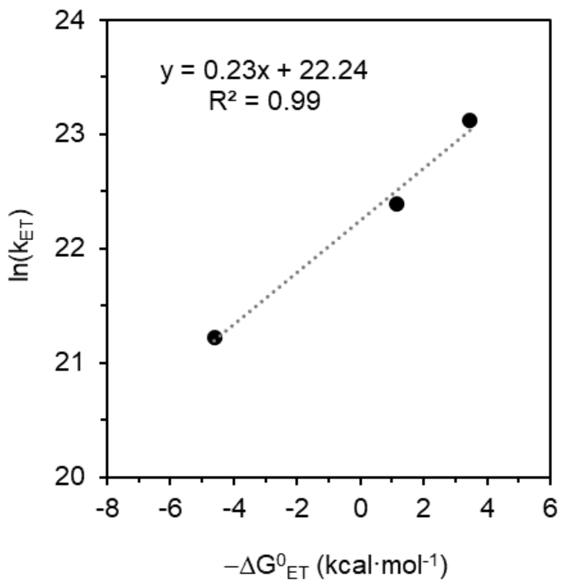

Figure S22. Plot of rate−driving force relationship for the photo-oxidation of alcohol substrates (benzyl alcohol, 1-phenylethanol and isopropanol) by $[Fe(pzTp)(CN)_3]^-$, using the kinetic data (k_{ET}) from the Stern-Volmer plots and the driving force calculated from the ΔE^o using the redox potential of the $[Fe(pzTp)(CN)_3]^-$ excited state (1.68 V) and the values for the oxidation of the different substrates determined by cyclic voltammetry. The error associated to the slope is ± 0.026.

Error estimation:

The rate−driving force analyses involves generating line of best fit and then interpreting the slope (*m*) of that line in order to extract mechanistic information. The line of best fit was generated using Excel and the LINEST function was used to determine the error in the slope (m_{err}) and was propagated to calculate the error in the Brønsted parameter calculated from the slope.

Cyclic voltammetry of alcohol substrates:

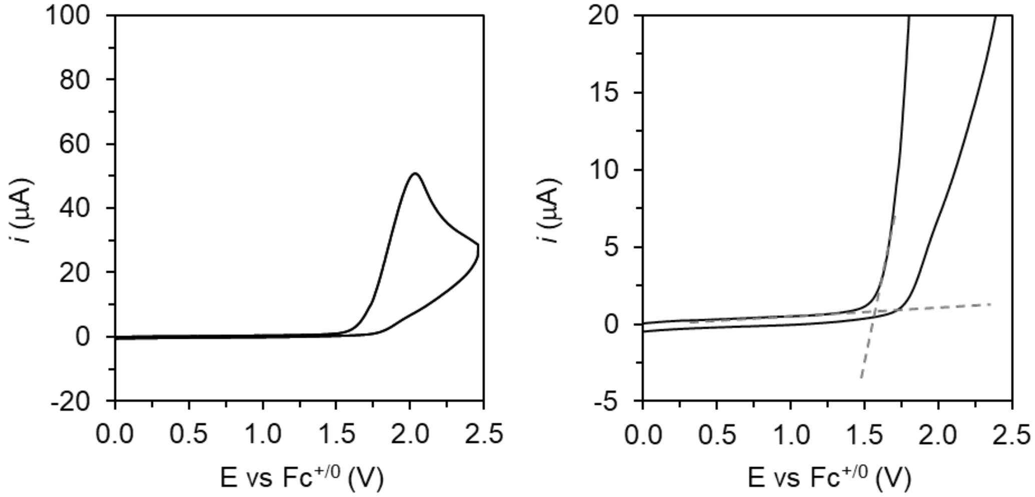

Figure S23. Cyclic voltammetry at 100 mV/s of a CH_3CN solution containing 0.1 M [^{n}Bu_4N][PF_6] as electrolyte and 20 mM benzyl alcohol using glassy carbon as working electrode, Pt disk as counter electrode, and Ag/AgOTf as reference electrode. The redox potential for the oxidation of the substrate was estimated from the onset potential observed in the CV.

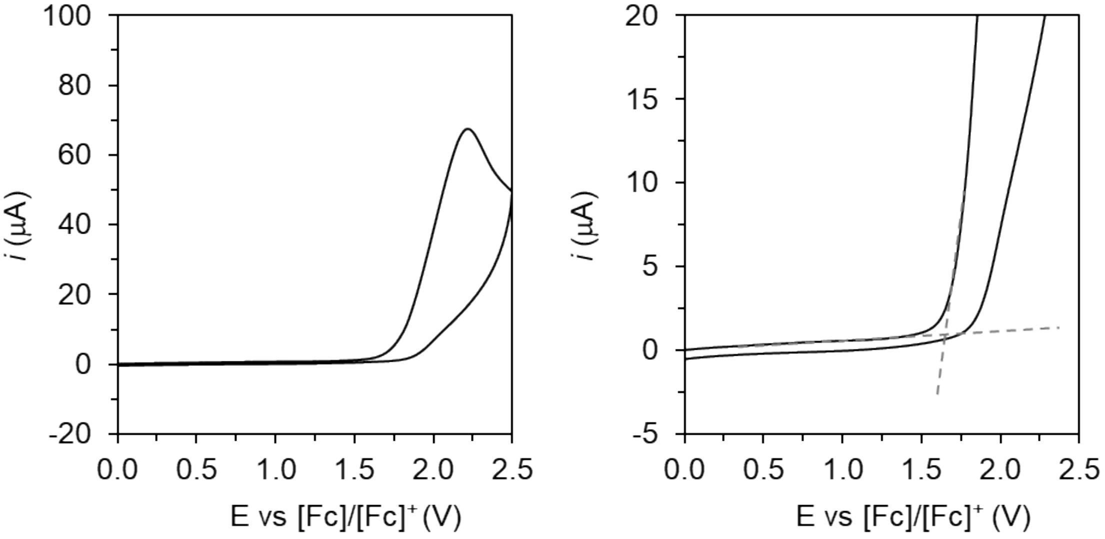

Figure S24. Cyclic voltammetry at 100 mV/s of a CH_3CN solution containing 0.1 M [^{n}Bu_4N][PF_6] as electrolyte and 20 mM 1-phenylethanol using glassy carbon as working electrode, Pt disk as counter electrode, and Ag/AgOTf as reference electrode. The redox potential for the oxidation of the substrate was estimated from the onset potential observed in the CV.

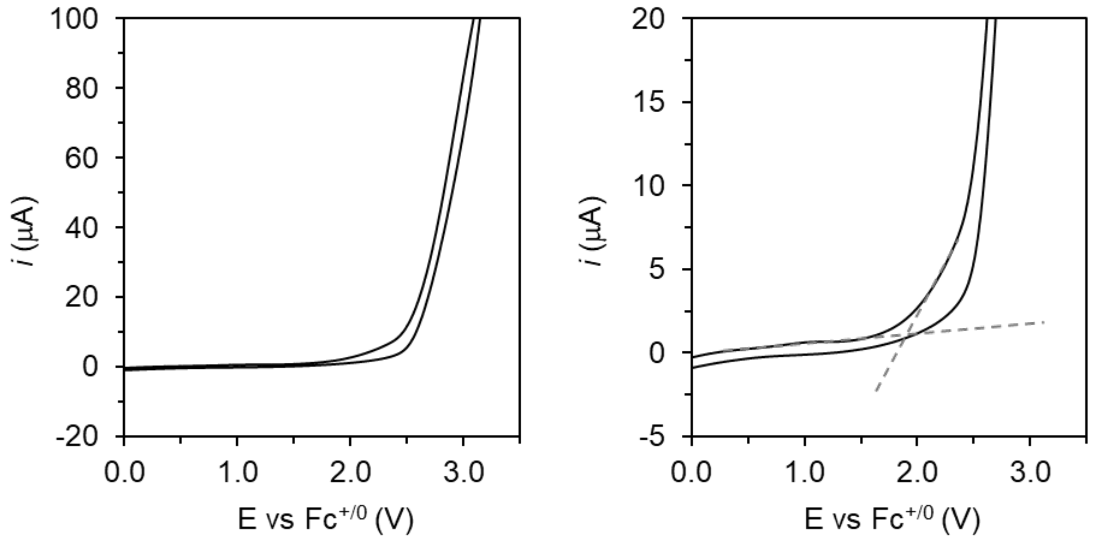

Figure S25. Cyclic voltammetry at 100 mV/s of a CH_3CN solution containing 0.1 M [^{n}Bu_4N][PF_6] as electrolyte and 20 mM isopropanol using glassy carbon as working electrode, Pt disk as counter electrode, and Ag/AgOTf as reference electrode. The redox potential for the oxidation of the substrate was estimated from the onset potential observed in the CV.

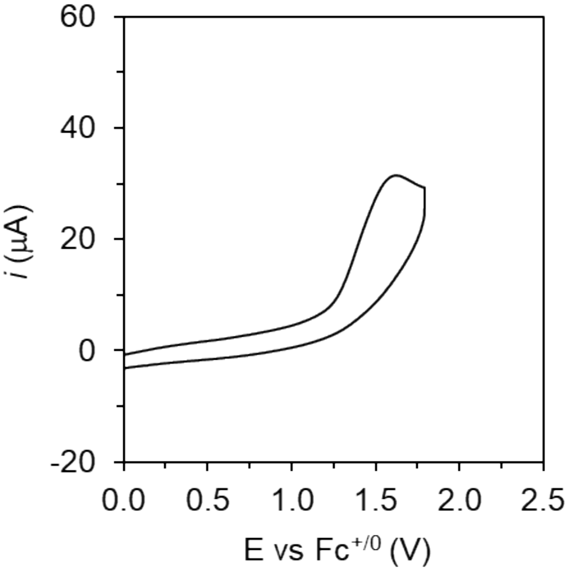

Figure S26. Cyclic voltammetry at 100 mV/s of a CH_3CN solution containing 0.1 M [^{n}Bu_4N][PF_6] as electrolyte and 5 mM of the ligand using glassy carbon as working electrode, Pt disk as counter electrode, and Ag/AgOTf as reference electrode.

8. Geometries

$[Fe(pzTp)(CN)_3]^-$ - doublet state

Fe	14.91833528648318	2.39504405277688	10.35024263472314
B	12.44690817712585	0.54522797757078	10.78864621190700
N	14.94149164821102	0.58996566007962	11.14375222182911
N	13.78108489758744	-0.10059515280889	11.25778842304126
N	13.64939483006468	1.67200277120870	8.96000105648208
N	12.60056763787349	0.86895011835705	9.28850122662097
N	13.32155180516081	2.79729931666510	11.45548285412386
N	12.29959171419956	1.90681223331382	11.52408353270185
N	16.83565727755315	3.50627249590033	12.47894802910926
N	17.34990073320617	1.67193169285622	8.60143698627836
N	14.77296490613468	5.20538323934337	9.08892388561471
N	11.29679216768674	-0.37359712353073	11.15241224038645
N	10.60710236244048	-1.11812775221606	10.24350476744405
C	15.92592387755748	-0.19640003406017	11.58582856703604
H	16.94299911028020	0.15949234067193	11.58533789921986
C	15.39888890795966	-1.42599934271987	11.98819381568811
H	15.93292038730373	-2.27121731168045	12.38873421448417
C	14.03416883926419	-1.32509995539331	11.76134515123621
H	13.23963029704081	-2.03361164850818	11.92485861996401
C	13.60018475207760	1.87628681244347	7.64514726270545
H	14.34527295994424	2.48986623754354	7.16603773479113
C	12.50106276879908	1.20212794186208	7.10091353983161
H	12.19460204795387	1.17061591883336	6.06872946080213
C	11.89153412644204	0.57716719589778	8.17454810167788
H	11.02735574520873	-0.05850175429979	8.24143723011908
C	12.94327328248444	3.89781305610924	12.10919201483592
H	13.61078312159986	4.74078033375744	12.17915727617853

C	11.64696337913074	3.73004129694519	12.60243133527803
H	11.06168369972043	4.43468588211292	13.16893211727022
C	11.27223908820622	2.45643289270383	12.20348885525542
H	10.35460910705007	1.91549815132411	12.35895771177583
C	16.12510812496544	3.09639687653831	11.65582956224682
C	16.44388390807747	1.96211984318494	9.26778634006122
C	14.84676263363228	4.15120401585686	9.57082126543747
C	10.90991814557523	-0.68963204153079	12.42080761786554
H	11.36369502081651	-0.22158167341202	13.27935005362023
C	9.93016069203103	-1.65350147457288	12.33576393706413
H	9.40199991346588	-2.12199514132139	13.15006200425381
C	9.78208182560753	-1.88061582464708	10.95266276581401
H	9.11345079407742	-2.56204212315531	10.44712147522462

$[Fe(pzTp)(CN)_3]^-$ - quartet state

Fe	15.06871811352482	2.45812738126246	10.50851901342180
B	12.45929541555083	0.55415931906379	10.76667711164931
N	14.98407794218962	0.56835625989372	11.12329670166150
N	13.80281346390071	-0.09048874890392	11.21921079813657
N	13.59049395007484	1.60902674082718	8.86514496031362
N	12.54229449815783	0.83432374335132	9.25462651492743
N	13.32789747885752	2.83352722454813	11.43716476855183
N	12.31531435032084	1.92878723742061	11.48264978528058
N	16.81841445368137	3.36754622166956	13.19047204272927
N	17.50960743810943	1.77062014499805	8.69980357225920
N	14.97908937389839	5.26642996604210	9.16622386209903
N	11.32742903662270	-0.36236947160047	11.19483442043876
N	10.68805179175323	-1.20813274946762	10.33855664559854
C	15.95420823982695	-0.28239545359124	11.47110034016073

H	16.98223252841965	0.04086394773054	11.47067762231677
C	15.39721786865619	-1.51928013822529	11.79892670527564
H	15.91258958035996	-2.40792678305640	12.12228935284689
C	14.03200838105382	-1.35782817418044	11.61769398545333
H	13.22042060099744	-2.05499153152156	11.74099218038697
C	13.45247980314917	1.82399535657763	7.56078794390746
H	14.18107311340632	2.42060883493730	7.03367405818442
C	12.29598773669416	1.18741210659235	7.08322207291291
H	11.91785403270572	1.17036034937092	6.07426531378460
C	11.74291441468190	0.57267812649598	8.19319568311005
H	10.86663692497579	-0.03935487103716	8.31028376522825
C	12.87869209501135	3.97113801448492	11.97498370946068
H	13.52749565226365	4.82877565050814	12.04317657620075
C	11.55026411568464	3.81266583441178	12.37186588943911
H	10.91130157103418	4.54208231438533	12.84042685833874
C	11.22727315454791	2.50944689829347	12.02820989687489
H	10.30315728583799	1.96720427887450	12.13310871811358
C	16.21452810838371	3.06118007957011	12.24527046457373
C	16.59661113707621	2.04910163237844	9.36000422568047
C	15.03051027196547	4.21366933491685	9.65203289956042
C	10.95289999533918	-0.61164862698844	12.48147251829961
H	11.37688952144755	-0.06223092192037	13.30628727020796
C	10.02792026138121	-1.63145408686950	12.46209930748285
H	9.52063886430370	-2.06977509031077	13.30598239802345
C	9.90131908035385	-1.96311693822666	11.09776270268559
H	9.27387835379897	-2.71419341270542	10.64022734442179

[Fe(pzTp)(CN)$_3$]$^-$ - sextet state

Fe	15.10128001006899	2.49290996334699	10.32488305775195
B	12.45522961641152	0.56045497951202	10.81490377503160
N	14.97033737793537	0.52324684213994	11.16173869490541
N	13.77880551346881	-0.11032627247525	11.27980174831599
N	13.62549966647482	1.68372324129257	8.94187306748446
N	12.58437452187540	0.88644225701245	9.30750376239004
N	13.32896706301498	2.79588416828110	11.55369672743845
N	12.29437981114093	1.91684740862632	11.55497144548889
N	17.01557344696364	3.47460867527171	12.79698644124302
N	17.50457421974989	1.50693913806478	8.32783802944516
N	14.72809581340296	5.46646928102184	8.99394236015129
N	11.30343822917903	-0.36622962843022	11.16395974868072
N	10.78747594089089	-1.26041817751522	10.27318710244775
C	15.91489811320486	-0.31200113489076	11.59765111759794
H	16.94930010105839	-0.00648352038606	11.59496193348676
C	15.33208448223564	-1.51614327237689	12.00463309218318
H	15.82544576443954	-2.38707732719631	12.40259812836243
C	13.97409493656886	-1.34776775083762	11.78316344316139
H	13.14825748374948	-2.01952651705985	11.94692477994857
C	13.51431224582004	1.90662606604839	7.63174731698805
H	14.24408254582562	2.51825301948005	7.12545886408870
C	12.38436185991523	1.25483639468612	7.13065560769711
H	12.02784070392657	1.23925825329788	6.11426153903416
C	11.81970422903914	0.62142811375933	8.22701069716817
H	10.95394696045109	-0.00945113923832	8.31936266957491
C	12.90288481885951	3.91509677480175	12.14122257553684
H	13.56934904901084	4.75642009604431	12.24670830575039
C	11.56559825231679	3.77400844329111	12.52428134729121
H	10.94015735556630	4.49696747522395	13.02076131808438

C	11.21392750886567	2.49560484371223	12.12240625696139
H	10.27656012907945	1.97230506796714	12.20246469247137
C	16.34517397393977	3.12911987676916	11.91341704860051
C	16.65836742746108	1.86816684245899	9.03690574438792
C	14.86453589542824	4.41349651455326	9.46510574946969
C	10.83092274552889	-0.61682402871665	12.41777295394600
H	11.15567384009338	-0.03870658522318	13.26707612207412
C	9.96353610129367	-1.68337548241493	12.33800337139021
H	9.40616967199197	-2.13372243448743	13.14302788830138
C	9.97487023871035	-2.04261286665367	10.97518489316067
H	9.42841233504137	-2.83154759876114	10.47914658250694

$[Fe(pzTp)(CN)_3]^{2-}$ - $^1A_1$ state

Fe	14.954970	2.416853	10.338134
B	12.469860	0.562129	10.788526
N	14.954047	0.584736	11.143554
N	13.789260	-0.097056	11.261384
N	13.666124	1.687128	8.954341
N	12.615680	0.883806	9.286793
N	13.328047	2.807794	11.456521
N	12.308554	1.914393	11.528686
N	16.785294	3.448490	12.579316
N	17.338040	1.631142	8.561784
N	14.738123	5.206817	9.063700
N	11.309739	-0.365446	11.150534
N	10.642983	-1.140415	10.252265
C	15.923079	-0.216956	11.581215
H	16.944071	0.127583	11.578210

C	15.383927	-1.449529	11.985480
H	15.910630	-2.302066	12.382010
C	14.023640	-1.334344	11.762141
H	13.220032	-2.033436	11.920988
C	13.598849	1.890458	7.641403
H	14.341687	2.504750	7.160093
C	12.489702	1.220916	7.101979
H	12.172139	1.192720	6.072424
C	11.889090	0.595344	8.179525
H	11.024612	-0.038997	8.251457
C	12.934140	3.904039	12.101333
H	13.595927	4.751807	12.166751
C	11.629033	3.734900	12.590761
H	11.035688	4.441195	13.147993
C	11.263450	2.460570	12.197182
H	10.346567	1.916414	12.344034
C	16.104275	3.066330	11.707560
C	16.441198	1.957131	9.239089
C	14.849200	4.147990	9.549398
C	10.916331	-0.662270	12.418815
H	11.356142	-0.169326	13.270169
C	9.952037	-1.645143	12.347059
H	9.424335	-2.104855	13.166860
C	9.821977	-1.903180	10.968442
H	9.170021	-2.605514	10.469290

9. References

(1) Suzuki, K.; Kobayashi, A.; Kaneko, S.; Takehira, K.; Yoshihara, T.; Ishida, H.; Shiina, Y.; Oishi, S.; Tobita, S. Reevaluation of Absolute Luminescence Quantum Yields of Standard Solutions Using a Spectrometer with an Integrating Sphere and a Back-Thinned CCD Detector. *Phys. Chem. Chem. Phys.* **2009**, *11* (42), 9850–9860. https://doi.org/10.1039/b912178a.

(2) a) F. Neese, *WIREs Comput. Mol. Sci.* **2012**, *2*, 73-78; b) F. Neese, *WIREs Comput. Mol. Sci.* **2022**, e1606.

(3) R. A. Marcus, *J. Chem. Phys.* **1956**, 24, 966. On the Theory of Oxidation−Reduction Reactions Involving Electron Transfer. I.
